# Polymer Brushes on Silica Nanostructures Prepared by Aminopropylsilatrane Click Chemistry: Superior Antifouling and Biofunctionality

**DOI:** 10.1021/acsami.2c21168

**Published:** 2023-02-11

**Authors:** John Andersson, Julia Järlebark, Sriram KK, Andreas Schaefer, Rebekah Hailes, Chonnipa Palasingh, Bagus Santoso, Van-Truc Vu, Chun-Jun Huang, Fredrik Westerlund, Andreas Dahlin

**Affiliations:** † Department of Chemistry and Chemical Engineering, Chalmers University of Technology, 41296 Gothenburg, Sweden; ‡ Department of Life Sciences, Chalmers University of Technology, 41296 Gothenburg, Sweden; § Department of Chemical and Materials Engineering, National Central University, Taoyuan 32023, Taiwan; ∥ R&D Center for Membrane Technology, Chung Yuan Christian University, Taoyuan 32023, Taiwan; ⊥ NCU-Covestro Research Center, National Central University, Jhong-Li, Taoyuan 32023, Taiwan

**Keywords:** polymer brushes, silica, antifouling, silanization, nanopores, nanochannels

## Abstract

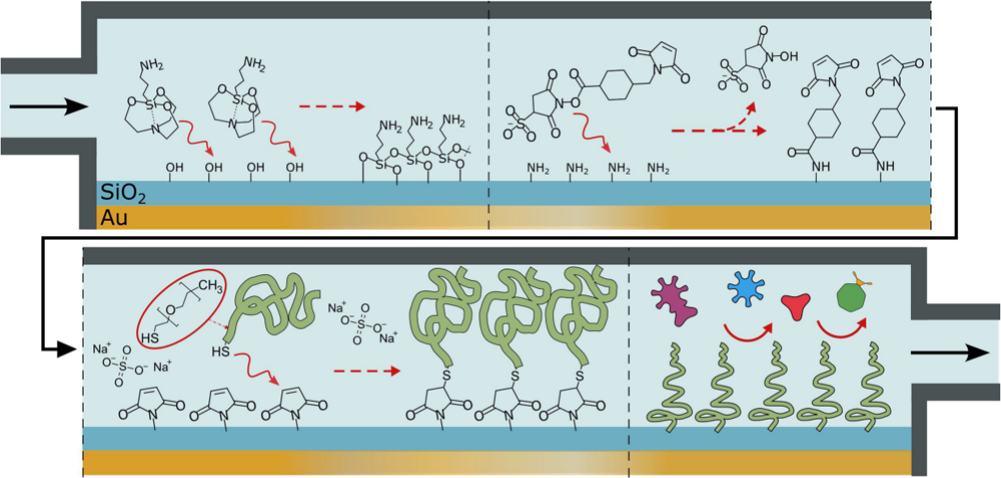

In nanobiotechnology, the importance of controlling interactions between biological molecules and surfaces is paramount. In recent years, many devices based on nanostructured silicon materials have been presented, such as nanopores and nanochannels. However, there is still a clear lack of simple, reliable, and efficient protocols for preventing and controlling biomolecule adsorption in such structures. In this work, we show a simple method for passivation or selective biofunctionalization of silica, without the need for polymerization reactions or vapor-phase deposition. The surface is simply exposed stepwise to three different chemicals over the course of ∼1 h. First, the use of aminopropylsilatrane is used to create a monolayer of amines, yielding more uniform layers than conventional silanization protocols. Second, a cross-linker layer and click chemistry are used to make the surface reactive toward thiols. In the third step, thick and dense poly(ethylene glycol) brushes are prepared by a grafting-to approach. The modified surfaces are shown to be superior to existing options for silica modification, exhibiting ultralow fouling (a few ng/cm^2^) after exposure to crude serum. In addition, by including a fraction of biotinylated polymer end groups, the surface can be functionalized further. We show that avidin can be detected label-free from a serum solution with a selectivity (compared to nonspecific binding) of more than 98% without the need for a reference channel. Furthermore, we show that our method can passivate the interior of 150 nm × 100 nm nanochannels in silica, showing complete elimination of adsorption of a sticky fluorescent protein. Additionally, our method is shown to be compatible with modifications of solid-state nanopores in 20 nm thin silicon nitride membranes and reduces the noise in the ion current. We consider these findings highly important for the broad field of nanobiotechnology, and we believe that our method will be very useful for a great variety of surface-based sensors and analytical devices.

## Introduction

In the wake of nanotechnological developments, the interdisciplinary field of nanobiotechnology, which brings the technological fruits of the nanotech world to bioscience, has been cited as one of the most promising new research areas since the advancement of biotechnology.^1−3^ This interest is also reflected in the industrial sector and in venture capital investments.^4^ The high potential impact of nanobiotechnology can be attributed to the new insights about the biological components of life obtained by utilizing novel nanomaterials, nanostructures, and supramolecular structures to study, manipulate, or interact with biomolecules at their native length scale.^1^ Additionally, new insights are offered by single-molecule analysis in contrast to ensemble measurements.^5^ When creating nanoscale devices, the surface-to-volume ratio increases drastically, and hence, the importance of surface interactions becomes paramount. Typically, there is a need to suppress interactions with the surface since the molecules to be studied or detected will be (or should be) in the solution phase. This is the case in, for example, electrostatic traps,^6^ nanochannels for DNA analysis,^7^ convex lens-induced confinement,^8^ and solid-state nanopore sensors.^9^ Notably, all these devices have oxidized silicon as the material forming the interface to the biological solution and preventing biomolecule adsorption is key to successful operation in all cases. Surface passivation of silica is also needed to direct the target molecules to the receptors in miniaturized affinity-based sensors that utilize, for instance, metallic nanoparticles on glass.^10,11^ Conversely, the possibility to functionalize silica surfaces with specific biological receptors on an otherwise inert background is also of interest for label-free sensing applications.^12^

Because of the dire need to passivate and functionalize silica, which extends also to medical purposes,^13^ several surface modification protocols have been developed. One option is lipid bilayers formed by spontaneous vesicle rupture.^14,15^ However, the antifouling performance of bilayers is often not sufficient, especially when handling biofluids, which has led to the development of other methods. An established protocol that has been commercialized is the block copolymer poly(l-lysine) with grafted poly(ethylene glycol) (PLL-g-PEG), which assembles spontaneously on oxide surfaces.^12,16−18^ However, PLL-g-PEG is a large molecule^19^ which complicates its use for modifying fine nanostructures, such as the interior of narrow channels or small pores. Furthermore, its attachment relies on electrostatic interactions with a negatively charged surface. Such noncovalent interactions can be broken, for instance at high salt content or low pH. The state-of-the-art in terms of antifouling properties is normally obtained with polymer brushes prepared by surface-initiated polymerization^20^ (grafting-from). Unfortunately, such protocols involve quite complicated synthesis schemes^21^ that cannot be easily adopted by researchers in the fields of molecular biology or biophysics. Also, harsh solvents are often needed which may not be compatible with commonly used micro- and nanodevice materials.^22^ Additionally, it is difficult to perform polymerization inside nanochannels due to limited reactant supply.^23^ An alternative is the direct attachment of polymers in solution onto surfaces^24^ (grafting-to), which can create repelling or selective coatings on metals.^25^ However, for silica, such protocols have so far almost exclusively relied on silanes (e.g., triethoxysilane groups), which are widely known to be unreliable due to their susceptibility to hydrolysis and self-polymerization. This results in nonuniform coatings on silica and poor reproducibility in the film properties.^26−30^ We note that Gidi et al. recently presented a protocol for passivating silica with PEG-silanes,^31^ but the antifouling performance was only evaluated with respect to a few selected biomolecules, not a real biological fluid like serum. In fact, for grafting-to methods there appears to be a trend of not thoroughly testing the antifouling performance, while this seems to be an established procedure when it comes to grafting-from.^20,32^ We also note that compatibility with nanochannels or nanopores is normally not demonstrated, neither for grafting-to nor grafting-from approaches.

**Figure 1:**
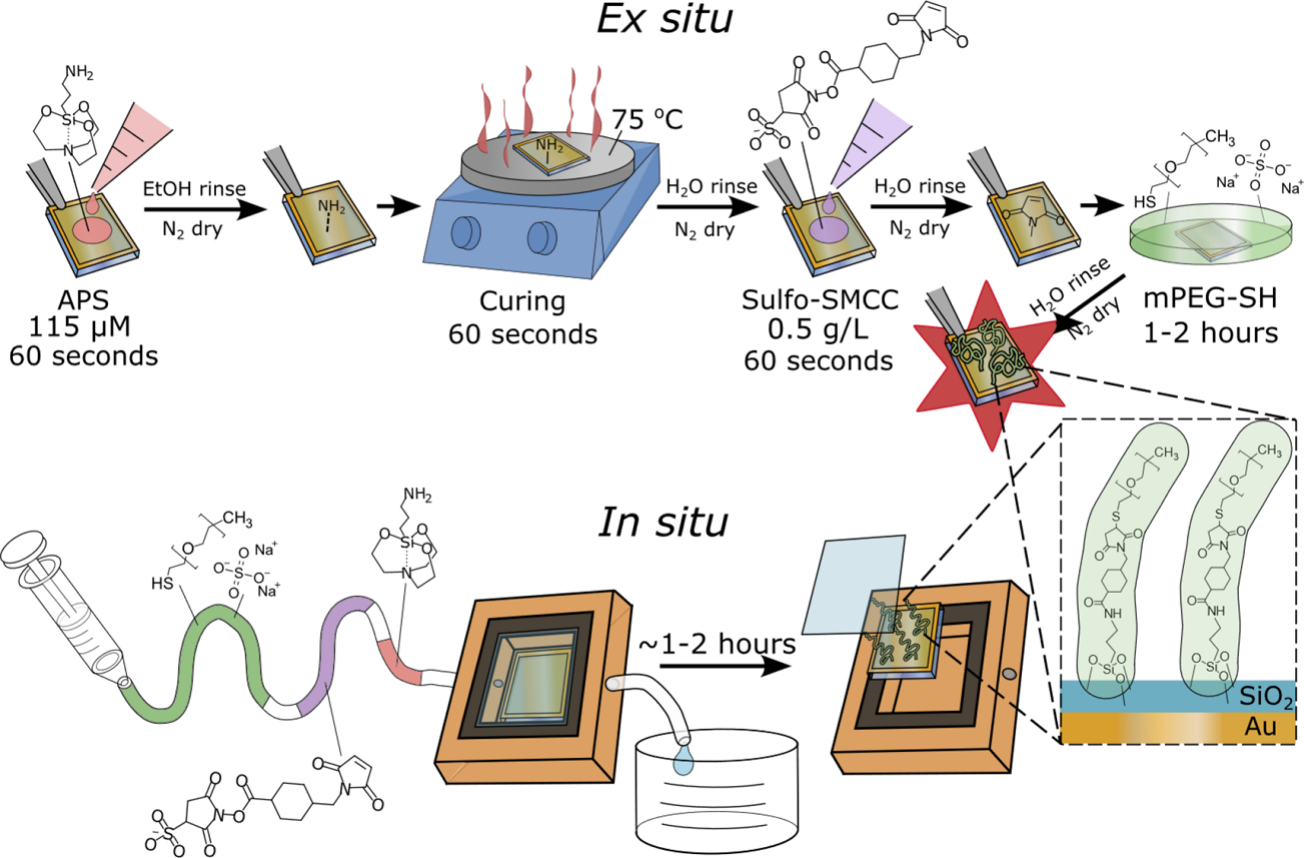
Method for silica passivation and/or biofunctionalization using silatranes, click chemistry, and PEG grafting. In the ex situ approach (top), the sample is immersed in different solutions. In the in situ approach (bottom), serial injections of the same three chemicals are performed without removing the sample.

A class of molecules that could overcome the limitations of conventional silanes is so-called silatranes.^33,34^ The closed and compact silatrane group is stable in the presence of water, while still being able to form covalent bonds with silica.^27,30,35^ Yet, to date silatrane compounds have not been used for grafting polymers to silica. In this work, we present a simple and generic method to make silica highly antifouling and/or bioselective, using aminopropylsilatrane and click chemistry for attaching thiolated poly(ethylene glycol) (PEG). This method does not require vapor-phase deposition, or harsh solvents. As illustrated in Figure 1, it can be performed by separate incubation steps (which we refer to as ex situ) or by serial injections (the in situ version). We show that the exact same thiol-PEG molecules used for direct grafting to gold^36^ also can be grafted to silica under identical conditions, and the resulting brushes are compared. The antifouling performance is evaluated by exposure to complete serum and shown to be superior to PLL-g-PEG and comparable to state-of-the-art even when including grafting-from methods. Furthermore, the silica surfaces can be made highly selective for biomolecule binding, which is shown by label-free detection of a protein spiked in serum with exceptionally high contrast. In addition, we successfully implement our method with different silica nanostructures, showing its great potential in nanobiotechnology.

## Experimental Section

The experimental section is available in the Supporting Information.

## Results and Discussion

### Surface Modification and Characterization Ex Situ

We first show the effectiveness of functionalizing SiO_2_ surfaces with polymer brushes using a simple immerse and rinse ex situ approach (Figure 1). The concentration of the 3-aminopropylsilatrane (APS) was 115 μM in ethanol, while the concentration of the cross-linker for click chemistry (sulfo-SMCC) was 0.5 g/L in 10× diluted PBS. The surfaces were characterized in the dry state using different techniques, in particular surface plasmon resonance (SPR). We noted that excessively high concentrations and incubation times of APS and/or sulfo-SMCC led to increasingly thick layers on the surface (Figure S1), most likely due to precipitation of APS (Figure S2) and the amphiphilic properties of sulfo-SMCC (Figure S3). However, when keeping the right concentrations and incubation times, the process was highly reproducible with a thickness variation for APS and sulfo-SMCC of merely ±0.1 nm according to SPR. The choice of solvent was also found to be important. In water, we could not produce APS layers thicker than 0.5–0.6 nm even at high concentrations and long incubation times (example with 460 μM APS and 1 h incubation in Figure S2), while the thickness is expected to be 0.7 nm based on the length of the molecule. This is most likely due to the high solubility of APS in water limiting its tendency to attach to the surface. We argue that previous work using APS in water to modify mica/glass^26,28,29^ has likely not achieved a complete monolayer. (Although the presence of APS on the surface was qualitatively confirmed, the surface coverage was not quantified in any way in those studies.) After binding, the APS layer should ideally be cured by heating to 75 °C to make it stable toward hydrolysis in water (Figures S4 and S5), similarly to films formed by silanes.^26,28−30^ Note that this refers to stability after surface binding: APS is perfectly stable in the presence of water while in the solution phase,^34^ in contrast to conventional silanes such as APTES. For sulfo-SMCC, hydrolysis may occur in aqueous environments,^37^ and the molecule is not easily soluble at high salt concentration, while the hydroxysuccinimidyl group requires a pH of 7–9 for efficient reaction with primary amines. Thus, while APS was stored in water, we always prepared a fresh sulfo-SMCC solution and opted for an intermediate ionic strength by using 10× diluted PBS buffer. The expected thickness of the layer is an additional 0.8–0.9 nm if the molecule is oriented vertically and has an amide bond formed with APS underneath (Figure 1).

**Figure 2:**
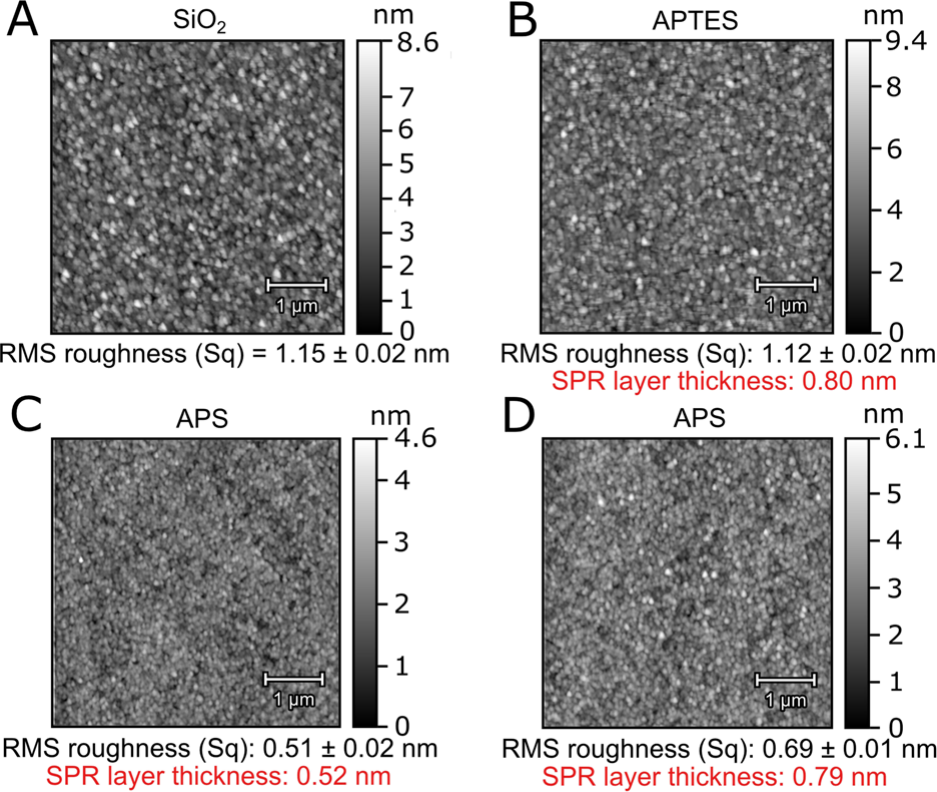
Surface roughness determined with AFM. (A) Typical height map of an SiO_2_ film deposited by ALD. (B) SiO_2_ functionalized with a monolayer of APTES (0.8 nm thickness measured in SPR). (C) SiO_2_ functionalized with less than a monolayer of APS (0.5 nm thickness measured in SPR), produced by immersing in PBS for 10 min prior to curing. (D) SiO_2_ functionalized with a full monolayer of APS (0.8 nm thickness measured in SPR). The provided root-mean-square (RMS) roughness value is the mean RMS of three separately measured 5 μm × 5 μm areas, with the error term denoting the highest measured difference from the mean.

Considering the clean SiO_2_ surface and the first modification step, we performed atomic force microscopy (AFM) to evaluate differences in homogeneity and surface roughness between APS and its silane counterpart APTES. By using silica-coated SPR sensors, we could determine the average optical layer thickness on the very same surface (see further below). We found a surface roughness of 1.15 nm for a clean 10 nm SiO_2_ thin film grown with atomic layer deposition (ALD) at 300 °C (Figure 2), in good agreement with the previously reported value of 0.985 nm at similar conditions.^38^ Even though APTES gave the same average layer thickness as APS, as expected^39,40^ (Figure S6), APS generated a much more smooth film, in line with previous observations for silanes versus silatranes.^26,27,35,41^ Remarkably, the surfaces with an APS monolayer even had a lower surface roughness (0.69 nm) than the bare silica (Figure 2). This “smoothening” effect was even more noticeable at an APS coverage corresponding to less than a monolayer. Since the roughness of our surfaces is generally low, we assume that the thickness of a monolayer of APS or sulfo-SMCC should correspond well to the actual size of the respective molecule. However, it should be kept in mind that for very rough silica surfaces the thickness determined from surface-sensitive techniques (e.g., SPR) should be a bit more than the molecular size in order to correspond to a conformal coating.

**Figure 3:**
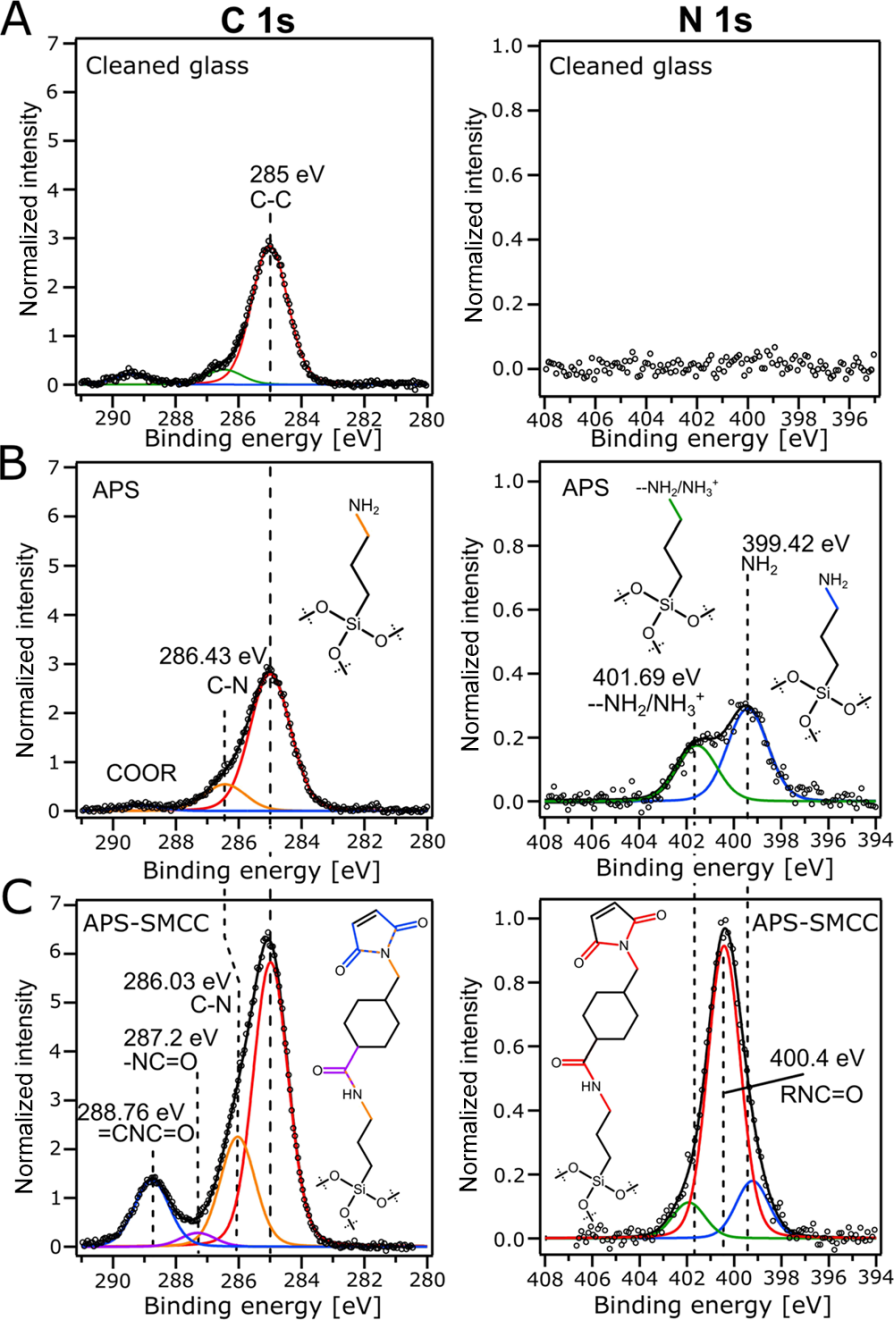
XPS measurements and peak fitting for the C 1s and N 1s peaks. (A) Cleaned borosilicate glass. (B) After APS. (C) After sulfo-SMCC. Black circles denote the measurement data points, and the black solid lines correspond to the measurement fit.

X-ray photoelectron spectroscopy (XPS) measurements were performed on ex situ functionalized borosilicate glass (predominantly SiO_2_) substrates to validate the presence of APS-SMCC. We observed a distinct peak in the C 1s spectrum around 285 eV binding energy, along with two small peaks at 286.5 and 289.5 eV, attributed to carbon contamination from exposure to atmosphere during transport and mounting and a lack of N 1s signal on the cleaned substrate (Figure 3A). After functionalizing the surface with APS, the C 1s spectrum in Figure 3B was similar to that for the cleaned substrate with a minor contribution at 286.43 eV attributed to C–N. Also, a clear signal appeared in the N 1s spectrum showing (at least) two peaks, which we attribute to freely available amines (−NH_2_) and hydrogen bonded (--NH_2_) or protonated (−NH_3_^+^) amines (Figure 3B), in agreement with observations of APTES-functionalized SiO_2_.^40,42−46^ However, Heinig et al.^30^ observed a single -NH_2_ peak for XPS measurements on polished silicon wafers (0.2 nm RMS roughness) incubated with APS in water for 30 min. We point out that the amount of --NH_2_ and −NH_3_^+^ in relation to −NH_2_ is expected to vary in the dry state depending on available surface charges and hydroxyl groups. Indeed, the intensity distribution between the N 1s peaks corresponding to −NH_2_ and --NH_2_/–NH_3_^+^ has been found to vary for APTES, depending on the substrate material,^42,47^ deposition method^42,45^ and aging.^42^ It is reasonable to assume that the same is true for APS layers, as the fully surface bonded molecules should be structurally identical. After incubation with sulfo-SMCC, both the C 1s and N 1s signals are substantially altered (Figure 3C). The C 1s peaks corresponding to C–C and C–N bonds are larger, and a new peak appears at 288.76 eV. Additionally, a large peak appears in the N 1s spectrum at 400.40 eV. These results are in excellent agreement with previous XPS studies on surfaces modified with sulfo-SMCC^48−51^ and both new peaks can be attributed to the addition of amide bonds. The small contribution at 287.2 eV is attributed to the carbon in the amide group formed after APS and SMCC have bound. Additionally, the S 2p region had barely any sulphonate (−C–SO_3_) signal^52^ from unreacted sulfo-SMCC (Figure S7), showing that the click chemistry worked as intended.

After verifying APS-SMCC formation by multiple techniques, we moved to the polymer grafting step. PEG was grafted using our previously published protocol for gold, where 0.9 M Na_2_SO_4_ is used to increase the grafting density.^36^ The attachment to sulfo-SMCC is via thiols reacting with maleimides, which enables us to graft the exact same thiol-PEG molecules (here 2 or 20 kg/mol) to gold and silica and investigate differences between the resulting brushes. A multiparameter SPR instrument was used to determine the optical thickness of all layers in their dry state with a resolution <1 Å by fitting the angular spectra with Fresnel models.^53−55^ Note that all SPR surfaces consist of gold, and the optical properties of the deposited SiO_2_ layer^56^ were characterized separately for inclusion in the Fresnel models (Figure S8). The bulk sensitivity and field extension are essentially unaltered when the SiO_2_ layer was 10 nm thin (Figure S9). A table describing optical parameters that were used for Fresnel modeling of each layer can be found in Table S1. Given their closely related molecular structure, we assume that the refractive index (RI) of APS is equal to that of APTES (1.42 from the CRC Handbook of Chemistry and Physics). We also assumed the same RI value for sulfo-SMCC to allow for a simple comparison of thickness.

**Figure 4:**
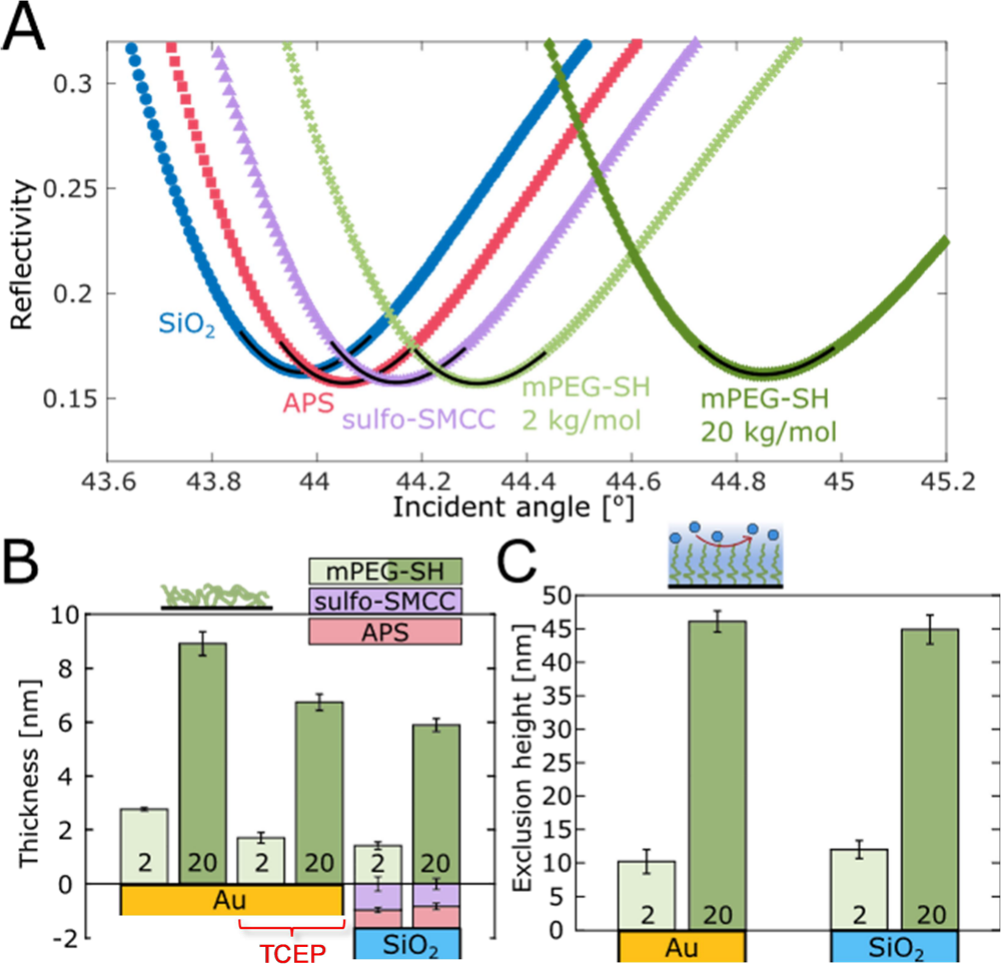
Verification of the ex situ modification by SPR. (A) Typical SPR minima in air of a clean SiO_2_ sample, after APS, after sulfo-SMCC, and after grafting thiol-PEG (either 2 or 20 kg/mol). Solid black lines show Fresnel model fits. (B) Summary of dry thicknesses obtained from fits to reflectivity spectra. In addition to silica, results are shown for direct grafting to gold^36^ (with or without TCEP), using the same 2 and 20 kg/mol thiol-PEG at identical conditions. Error bars are two times the standard deviation. (C) Exclusion heights^53^ of the final brushes measured in PBS buffer. Error bars represent the uncertainty of the measurement mean within a 99% confidence interval.

Figure 4A shows the typical angular SPR spectra during the ex situ procedure. The thicknesses of the APS and sulfo-SMCC layers (Figure 4B) obtained from fitting were in excellent agreement with the sizes of the different molecules, confirming that monolayers have been formed. To verify that neither sulfo-SMCC nor thiol-PEG physisorbs to the SiO_2_ surface to any comparable extent, control measurements were made by excluding the APS step. The lack of a significant SPR signal in this case (Figure S10) confirms covalent bonding via click chemistry and negligible amounts of physisorbed PEG. SPR was also used to compare the dry and swollen brush thickness by determining the so-called “exclusion height”, which represents the characteristic distance from the surface at which a macromolecule (e.g., a protein) cannot approach further.^53−55^ This parameter was obtained by utilizing the total internal reflection (TIR) angle to obtain the bulk RI and Fresnel models.

When comparing the PEG brushes on silica with those on gold, we found that the hydrated brushes had the same heights for both molecular weights of PEG (Figure 4C). However, when comparing the dry layer thicknesses, the amount of PEG was lower on SiO_2_ (Figure 4B) and the grafting densities were 0.51 and 0.19 chains/nm^2^ for 2 and 20 kg/mol PEG, respectively. Thus, directly after the grafting, there was more PEG on gold than on silica, but it did not contribute to the extension of the polymer brush. Although the brush extension in a good solvent depends very weakly on grafting density^36^ (to the power of 1/3), a minor difference in the exclusion heights should be notable. As we have previously shown,^53^ additional polymers adsorbed to the solid surface underneath the brush (i.e., not end-grafted) can give rise to this effect. While we did not detect any binding of non-thiolated PEG to gold using SPR (Figure S11A), we consistently observed that a large fraction (∼40%) of the thiol-PEG was in fact reversibly bound, as it slowly desorbed in water (Figure S11B). We emphasize that this effect was never observed for silica modified according to the protocol developed here. Still, to further elucidate why physisorption of thiol-PEG occurred on gold but not silica, we included tris(2-carboxyethyl)phosphine hydrochloride (TCEP) at a concentration of 25 mM in the grafting solution, which cleaves disulfides that may have formed. The PEG amount on gold was then strongly reduced and became very similar to that on SiO_2_ (Figure 4B), strongly suggesting that it was PEG chains linked by disulfide bonds that were physisorbing on gold. Note, however, that TCEP *must not be used* when grafting to APS-SMCC on silica because it interferes with the reaction (further discussion in the Supporting Information).

### Monitoring the Modifications in Situ

**Figure 5:**
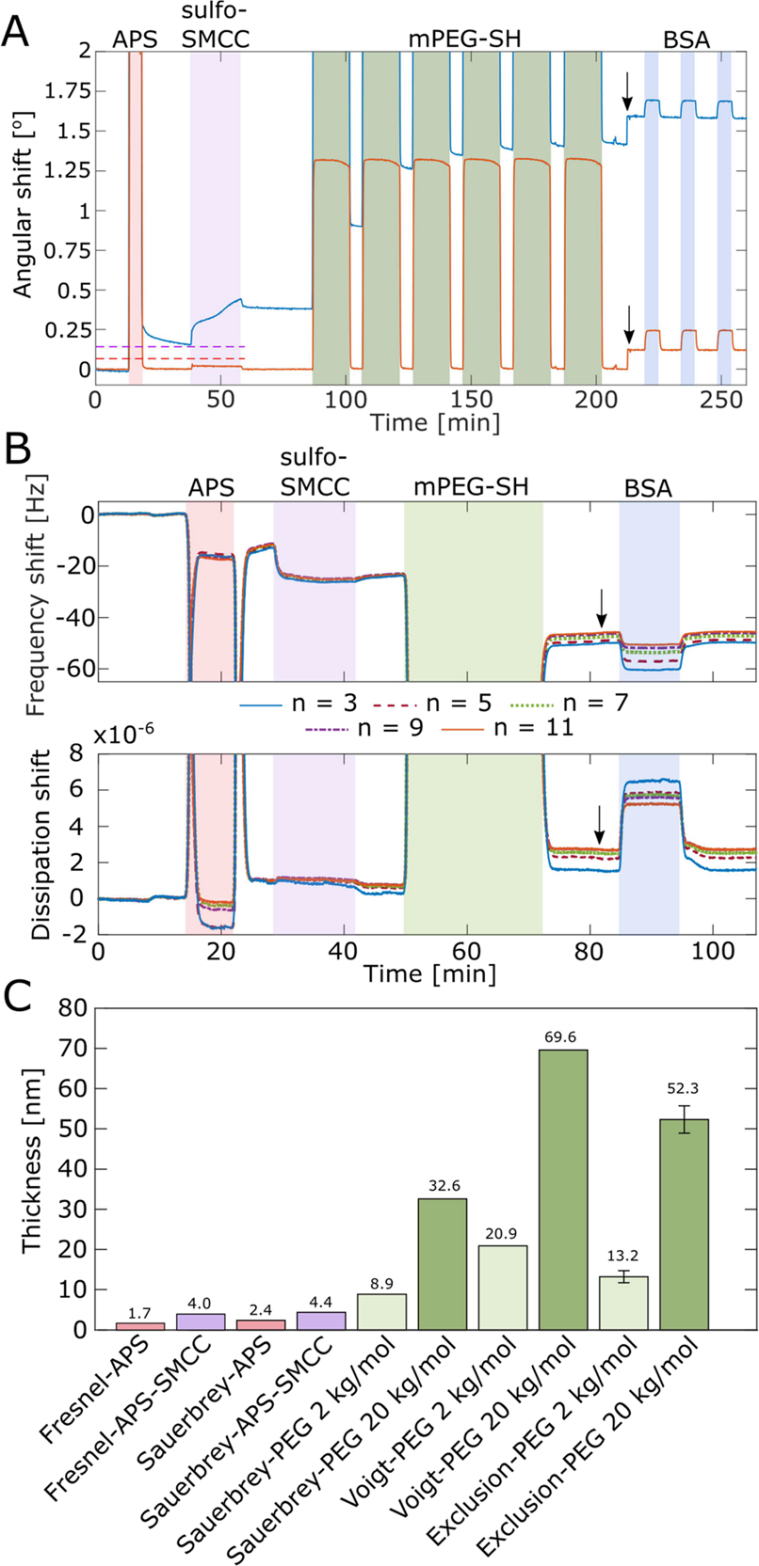
Monitoring in situ functionalization of APS, sulfo-SMCC, and PEG. (A) SPR data of injections of APS (460 μM in ethanol), sulfo-SMCC (2 g/L in 10× diluted PBS), and multiple injections of 20 kg/mol thiol-PEG (0.12 g/L in 0.9 M Na_2_SO_4_). The SPR angle (blue trace) and TIR angle (orange trace) responses are shown. The running buffer is 10× diluted PBS until the black arrow, where it changes to regular PBS. The final BSA injections (10 g/L) confirm a repelling brush and are used to determine exclusion heights. The dashed lines correspond to the theoretical responses from 0.7 nm APS and an additional 0.9 nm sulfo-SMCC according to Fresnel models of nonhydrated films (RI 1.42). (B) Same experiment but in QCMD with one injection of 2 kg/mol thiol-PEG (1 g/L in 0.9 M Na_2_SO_4_). (C) Summary of total thicknesses vs the SiO_2_ surface measured for the in situ modification by different methods and models in SPR (Fresnel or exclusion height) or QCMD (Sauerbrey or Voigt).

We now move to the in situ protocol, which is useful if the sample should ideally not be dismounted, dried, or heated to 75 °C. Real-time SPR measurements of the APS-SMCC-PEG functionalization procedure on clean SiO_2_ are presented in Figure 5A for 20 kg/mol PEG. (Corresponding data for 2 kg/mol PEG in Figure S12A.) Both the SPR and TIR angular traces are displayed to clarify when a substantial liquid bulk RI change occurs, an effect which is particularly clear for APS as it is introduced into ethanol. Clear binding of APS, sulfo-SMCC, and PEG is detected throughout the functionalization procedure. At the end, no binding of 10 g/L BSA occurs, showing that a repelling polymer brush layer has been formed. As a control, using PEG chains with thiol groups in both ends did not yield repelling coatings (Figure S13). Still, with the in situ method it does become more difficult to create perfect monolayers of APS and sulfo-SMCC because care must be taken when it comes to concentrations and incubation times. We typically used ∼5 min for the APS injection and ∼15 min for sulfo-SMCC for successful results. However, these are approximate times because the liquid exchange of the system comes into play. Ideally, the different solutions above the surface should be changed quickly without mixing bulk components, a feature which is most easily achieved with microfluidic technologies and appropriate design of the liquid cell. Also, it must be kept in mind that since the APS layer cannot be cured when using the in situ protocol, it slowly starts desorbing after introducing an aqueous solvent (as can be seen in Figure 5A). Fortunately, binding of sulfo-SMCC had a stabilizing effect on the layer and removed the need for curing as long as it was introduced reasonably fast. For instance, Figure 5A shows that there is no equilibrium established before, during or after the injection of APS, but the baseline does stabilize after rinsing away excess sulfo-SMCC. The protecting effect from sulfo-SMCC can be explained by the longer chain length of the conjugate imposing a steric hindrance for hydrolysis at the SiO_2_^57^ and the fact that the surfaces became more hydrophobic (Figure S14). Furthermore, we never observed any desorption of the final PEG brushes, even when they were exposed to high shear flows and surfactants.

To further characterize the in situ modification, we used quartz crystal microbalance with dissipation monitoring (QCMD) at several harmonics (Figure 5B). The resulting shifts in frequency and dissipation confirm binding, and the increase in dissipation shows that a viscoelastic layer has been added to the surface.^53^ (See also data for 20 kg/mol PEG in Figure S12B.) As an alternative to the exclusion height determined by SPR, the acoustic thickness of the brush was modeled using the QCMD data. The layer was either treated as rigid, using the linear Sauerbrey relation, or as viscoelastic, using the multiparameter Voigt model.^53^ While the Sauerbrey constant (17.5 ng/cm^2^ per Hz) accurately determines contributions from rigidly attached adsorbates, it will generally underestimate the thickness when the frequency signal is dampened by the viscoelastic properties of the adsorbent and solvent.^58^ The Voigt model instead assumes that the film is viscoelastic and yields an acoustic thickness that may be interpreted as the boundary at which an acoustic shear wave no longer is influenced by the hydrated film.^59^ A limitation of both models is that they assume a homogeneous layer, while the stretched polymer brushes adopt a parabolic density profile.^36^ Note that both models are conceptually different from the exclusion height measured by SPR.^53−55^

Considering the different thickness values (Figure 5C), the acoustic thickness of APS calculated using the Sauerbrey eq (2.4 nm) is similar to that for the sulfo-SMCC layer (increase to 4.4 nm). Both values are in excellent agreement with the SPR thickness after similar incubation times. Still, we emphasize that the exact thicknesses of both these layers will depend on the incubation times (equilibrium is not established) and that the layers are thicker than those obtained with the ex situ method, which gives perfect monolayers (to the extent we can measure). The acoustic thicknesses and the exclusion heights of the PEG brushes show similar trends as we have observed on gold.^53^ In particular, the higher Voigt value is expected and may be attributed to the parabolic density profile: the chains are dynamic and at any point in time, a few will temporarily extend longer than others simply due to fluctuations. Interestingly, the Voigt thickness on SiO_2_ is similar to what we previously measured^53^ (64 nm) for 20 kg/mol PEG brushes on gold, while the Sauerbrey thickness differs considerably compared to the value in that study (45 nm). This is likely related to the extra PEG present on gold, as mentioned above (Figure 4C) because TCEP has not been added during grafting in our previous work. It can also be seen that the exclusion heights are slightly higher for the in situ method, which can be at least partly attributed to the slightly thicker APS-SMCC layers underneath.

### Antifouling Tests

To thoroughly test the surface passivation, we performed liquid injections of a biofluid (bovine serum) and measured the amount of adsorbed proteins after rinsing, which is a common method to quantify antifouling in bioanalytical settings.^20,32^ Given that the response is linear, a relative resistance to fouling can be easily calculated by comparing with the protein amount on an unmodified surface exposed to biomolecules in the same manner. To get absolute values of bound amounts, optical techniques such as SPR are preferable for accurate quantification with high resolution.^36,53^

**Figure 6:**
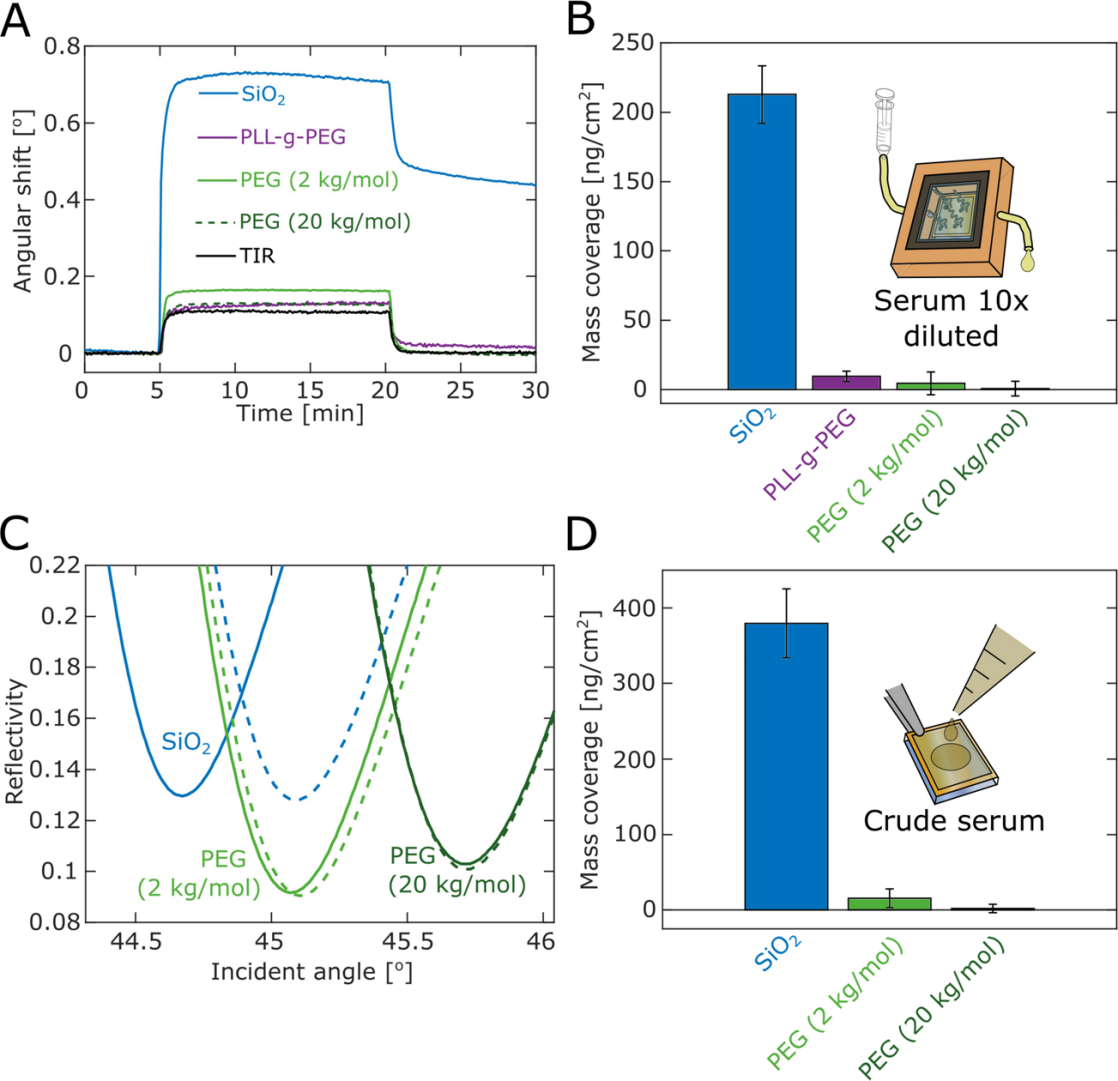
Quantifying antifouling properties. (A) Example SPR sensorgrams when introducing diluted adult bovine serum (for 15 min) on clean SiO_2_, PLL-g-PEG, and APS-SMCC-PEG. The black trace is an example of the TIR angle signal, which shows when the bulk molecules have been fully rinsed away. (B) Summary of SPR in situ signals from adsorbed serum biomolecules on the different surfaces. The amount of proteins adsorbed is determined from the remaining signal in the SPR angle after rinsing (calculation in the Supporting Information). (C) SPR spectra in air before (solid) and after (dashed) exposure to crude bovine serum for 20 min, rinsing in water and drying. (D) Fouling levels from the crude serum quantified by Fresnel models. (A RI of 1.52 and a density of 1.33 g/cm^3^ was assumed for the adsorbed biomolecules.) Error bars represent sample to sample variation. On some surfaces, the fouling was less than the experimental detection limit.

For in situ measurements, the serum was diluted (10× in PBS) and filtered (0.2 μm) simply to enable flow through the SPR system. To compare our method with an established (and commercialized) option designed to make negatively charged surfaces antifouling, we also modified silica with PLL-g-PEG. We estimated a surface coverage of 67 ng/cm^2^ for PLL-g-PEG (Figure S15A), which was 50% of that of our thiol-PEG (excluding APS-SMCC) when accounting for differences in RI increment (0.158 cm^3^/g for PLL-g-PEG^12^ and 0.134 cm^3^/g for PEG^36^). For the exact same PLL-g-PEG construct, previous studies have measured comparable values (e.g., 96 ng/cm^2^ on sputtered silica films^60^) while the block copolymer assembles more densely (∼160 ng/cm^2^) on metal oxides due to their higher negative charge.^17^ We also noted that due to its noncovalent grafting, the PLL-g-PEG brush was not stable if exposed to high flow rates or surfactants (Figure S15B). We show data obtained for serum injected directly after forming a saturated layer of PLL-g-PEG. Figure 6A shows sensorgram traces when rinsing out serum after equilibrium establishment, and Figure 6B summarizes the SPR signals corresponding to irreversible adsorption. The resistance was found to be 95.5% for PLL-g-PEG (carrying 2 kg/mol PEG chains) and 98% for our 2 kg/mol PEG brushes. In absolute numbers, the latter corresponds to ∼4 ng/cm^2^ and is considered as “ultralow” fouling.^32^ For 20 kg/mol PEG, the resistance reached an exceptionally high value of 99.7% (<1 ng/cm^2^ fouling). We note that the serum resistance of PLL-g-PEG achieved here is slightly poorer than initial reports, who measured a few ng/cm^2^.^16,17^ However, those studies were not using silica surfaces, but metal oxides. Although such surfaces are highly relevant for certain applications (e.g., TiO_2_ for implants^18^), our work is focused on silica because it is the material found in so many novel nanostructured analytical devices. Hence, we conclude that our method is superior to PLL-g-PEG for making silica antifouling, even for planar surfaces. In addition, we also investigated the antifouling performance of gold modified with a standard dextran matrix coating. SPR experiments conducted in the same manner as in Figure 6A gave quite high fouling (Figure S16).

To test the antifouling performance even further, we exposed SiO_2_ surfaces prepared ex situ to crude serum (undiluted and unfiltered) for 20 min and quantified the adsorbed amount of biomolecules by SPR spectra in air (Figure 6C) after rinsing with water. Our brushes exhibited low fouling also after this treatment (Figure 6D), though a minor increase in binding was observed for the 2 kg/mol PEG brushes (15 ng/cm^2^ on average). Still, the 20 kg/mol PEG gave a fouling level comparable to the uncertainty of the measurement (<2 ng/cm^2^ on average). These results clearly challenge the view that simple PEG coatings prepared by grafting-to are not competitive for truly efficient antifouling.^20^ Even for the case of (much thicker) polymer brushes prepared by grafting-from,^32^ the fouling is often at least a few ng/cm^2^ higher. Also, we again point out that such modifications are much more complex to perform and may not be compatible with all nanostructures.

### Biofunctionalization and Selectivity

**Figure 7:**
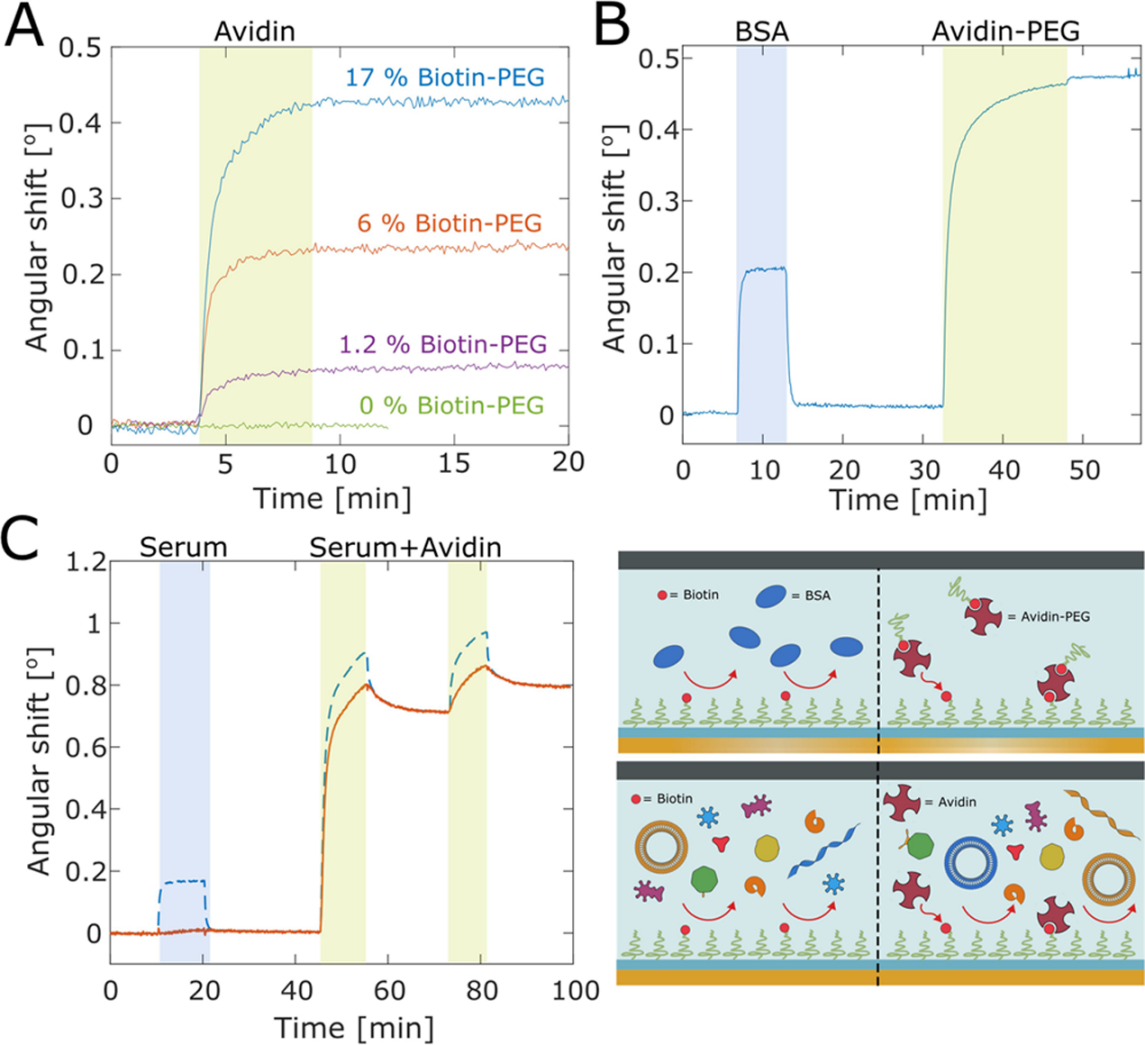
Selectivity test of silica modified with APS-SMCC-PEG-biotin in SPR. (A) Avidin binding for different fractions of biotinylated chains (during grafting). (B) Injections of BSA (10 g/L) and a PEG-biotin-avidin 1:1 conjugate (0.05 g/L in 95% PBS, 5% water). (C) Injection of serum (10× diluted) and serum spiked with avidin (0.1 g/L). The red trace has been corrected for the bulk response using the TIR angle,^61^ i.e., it corresponds to surface binding only.

For many applications, a repelling surface is not sufficient as there is a need to introduce receptors for selective biomolecule binding. Such extra chemical functionalization may reduce the antifouling performance.^32^ To demonstrate that our method also can be used to provide surface bioselectivity, i.e., the capability to capture a certain biomolecule with minimal nonspecific adsorption, we used PEG chains with biotin as the end-group (and thiol in the other). These brushes were produced by incubating the surface with a fraction of biotinylated PEGs, maintaining the same total mass concentration (in 0.9 M Na_2_SO_4_). The amount of avidin bound to the biotin on the surface increased with the fraction of biotinylated chains up to ∼17% (Figure 7A). The binding selectivity was first tested by injecting a high concentration (10 g/L) of BSA, which gave no significant signal except the bulk response during the injection^61^ (Figure 7B).

Often the introduction of biotin on the surface is done merely to enable further functionalization steps with avidin as a cross-linker to other biotinylated molecules. To show that our surfaces are compatible with such modifications, we also measured binding of an avidin-biotin-PEG conjugate (Figure 7B). This conjugate was produced from mixing avidin and thiol-PEG-biotin at 1:1 molar ratio (740 nM concentration for 5 h at room temperature). The clear binding illustrates that it is possible to immobilize biotinylated receptors on the inert PEG background for affinity-based sensing of practically any analyte.

We also injected serum on the biotinylated surfaces (Figure 7C), which led to extremely low binding (>98% resistance), thereby showing that introducing a fraction of biotinylated PEGs does not significantly reduce the antifouling performance. In addition, upon injection of serum with avidin spiked at 0.1 g/L, clear binding was observed, showing that our silica surfaces are capable of highly selective analyte capture in complex media. For comparison, in previous work aiming to detect avidin from serum, the signal from nonspecific binding was sometimes comparable to that of avidin, leading to strongly reduced sensor performance.^11^ The signal from avidin is slightly higher when binding occurs from serum, which is because avidin interacts with other serum proteins due to its high positive charge and “drags” them to the surface. This is a well-known effect, not the least for BSA, which is negatively charged and the most abundant protein in bovine serum. We could, for instance, see that BSA did adsorb to the surface after avidin had bound (data not shown).

Besides proving highly specific analyte capture, the results in Figure 7 illustrate that the bottleneck when using label-free surface-sensitive techniques for analysis in complex media really lies in the surface chemistry rather than the instrumental resolution.^62^ Even if we have reduced the nonspecific binding by more than 98%, a small false positive signal is still detectable (Figure 7C) because the signal-to-noise ratio is more than 100. This means that there is no point in improving the resolution of the instrument if the aim is to perform sensing in complex media. We also emphasize that the avidin-biotin system is not representative of biomolecular interactions in general due to its unusual high affinity, which causes great concerns when evaluating sensor performance across studies.^10^ Hence, we prefer not to discuss detection limits in terms of concentration (or any other parameter), but rather focus on the extremely high selectivity of the binding, which to the best of our knowledge has never previously been demonstrated, at least not with a grafting-to method.

### Passivation of Nanochannels

**Figure 8:**
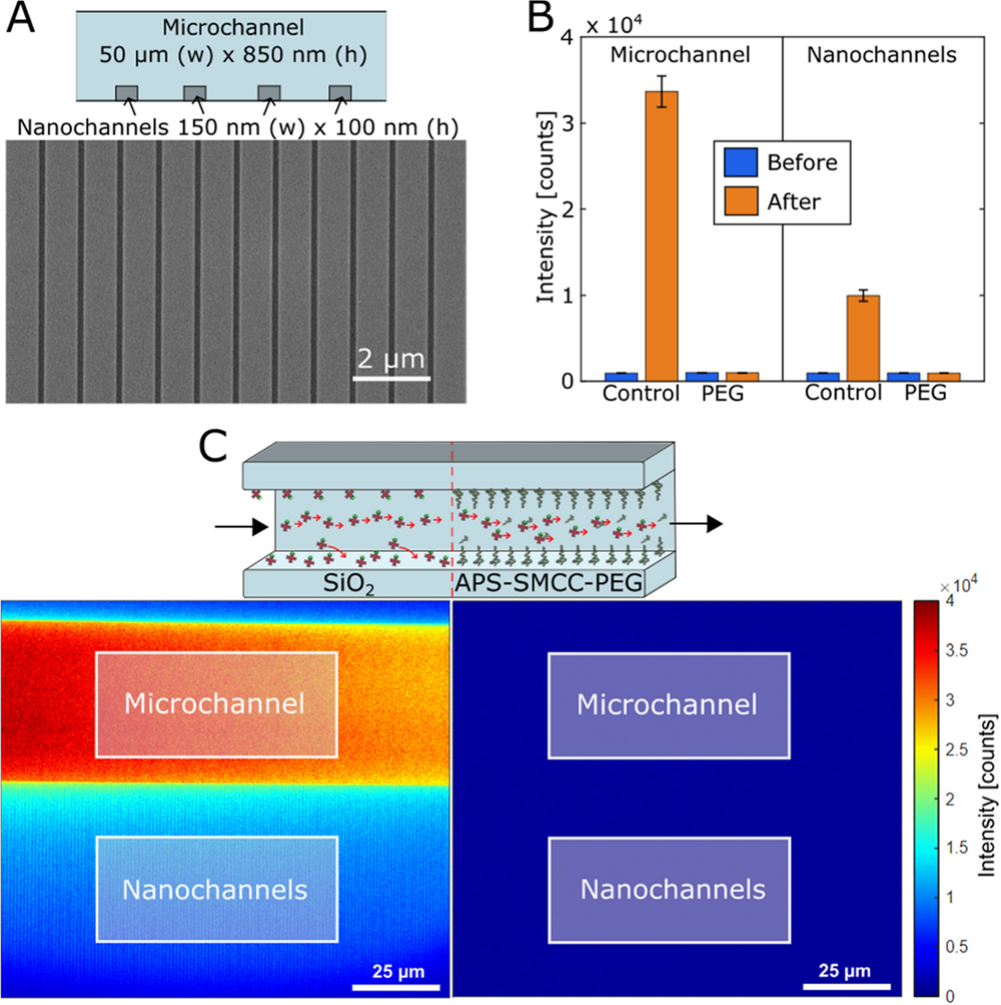
Surface modification of nanofluidic channels. (A) Chip design with parallel nanochannels connected to a microfluidic channel. The electron microscopy image shows the nanochannels from above. (B) Summary of intensities measured from channels before and after introducing avidin-FITC. Error bars represent the variation from subsequent acquisitions. (C) Fluorescence microscopy images of avidin-FITC adsorbed inside a nanofluidic chip made of SiO_2_. The regions where the intensities were measured are indicated. The right image was obtained in the same way but after passivation with APS-SMCC-PEG. (No intensity larger than the background is detected.)

To extend the range of applications for the APS-SMCC-PEG modification beyond planar surfaces, we applied the method to fluidic chips with micro- and nanosized channels in SiO_2_. Such chips are established for several bioanalytical purposes,^7^ in particular electrostatic trapping^63^ of biomolecules and stretching^64^ of DNA. The use of nanofluidic devices to study DNA-protein interactions at the single-molecule level has gained increasing interest^65^ and passivation of the channel surfaces is crucial to minimize adsorption of proteins.^14^ To quantify protein adsorption inside the channels, we used avidin conjugated with fluorescein (FITC) and fluorescence microscopy imaging. Note that native avidin has a particularly high tendency to stick to surfaces owing to its glycosylated groups and high positive charge in a wide pH range.^66^ We followed the APS-SMCC-PEG in situ passivation protocol using 2 kg/mol PEG, but with an additional ethanol rinse after introducing APS to fully remove excess molecules inside the channels. Rinsing with ethanol did not cause hydrolysis of the APS layer (Figure S17) and can thus be safely used to thoroughly wash the system. The functionalization was carried out by filling the inlet reservoirs with APS, sulfo-SMCC, or PEG solutions, while only loading the corresponding solvent or buffer in the outlet. The microchannel was first flushed for a few minutes, after which the solution was flushed also through the parallel nanochannels (Figure 8A).

The resistance of the modified SiO_2_ channels toward avidin-FITC was tested by comparing the mean fluorescence intensity to an unmodified chip after flushing 50 μg/mL avidin-FITC in PBS for at least 20 min and rinsing with PBS for 10 min. Note that the nanochannels only occupy a fraction of the projected area, so their intensity should not be compared to that of the microchannel. Instead, we evaluated the antifouling of each channel type separately in the same manner (Figure 8B). While avidin-FITC clearly adsorbed to unmodified silica (Figure 8C), no fluorescence at all could be detected from either the microchannel or the nanochannels after the PEG brush formation, in agreement with SPR results (Figure 7A). In other words, any protein adsorption was below the noise limit of the fluorescent readout, which was around 0.1% of the full signal from avidin binding to unmodified channels. To further confirm that the channels were not clogged, and that the protein was transported through efficiently, we detected fluorescence inside the channels during the protein injections (Figure S18A) and by the outlet (chip design in Figure S18B).

### Modifying Solid-State Nanopores

**Figure 9:**
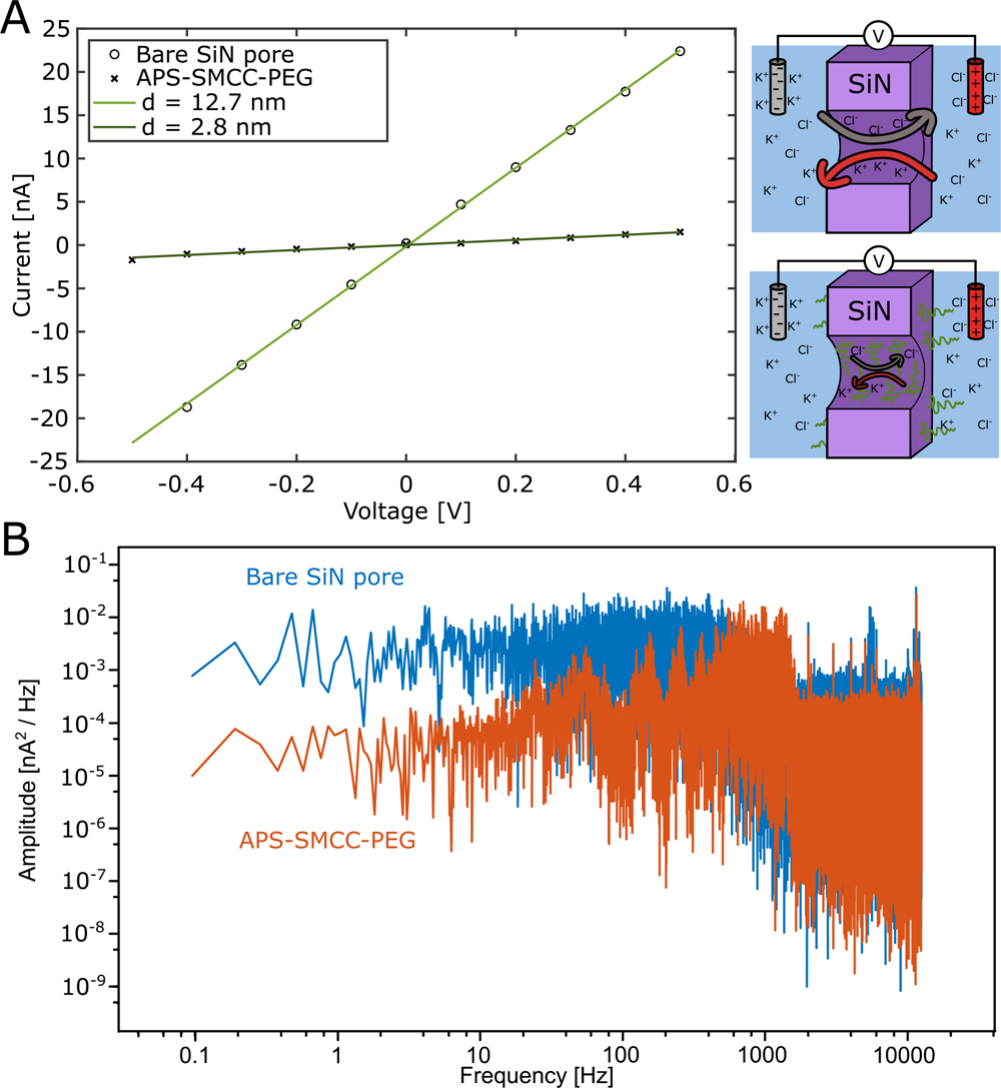
Surface modification of a solid-state nanopore verified by the ion current. (A) Typical voltage–current relations before and after grafting 2 kg/mol PEG to a pore in situ. The lines show the fitted conductance (46 nS before and 2.9 nS after) and corresponding pore diameters. (B) Power spectra for the same pore, showing reduced low-frequency noise after PEG grafting.

Finally, we also evaluated our method on solid-state nanopores in silicon nitride membranes, which are widely used for bioanalytical purposes.^9,15^ Single nanopores were prepared in silicon nitride membranes using controlled dielectric breakdown^67^ (Figure S19). The pores could be successfully modified using both the ex situ and in situ protocols and we noted no difficulties with wetting PEG-modified pores after they had been dried. The pore conductance changed considerably after forming 2 kg/mol PEG brushes, confirming successful modification of the silicon nitride (representative example in Figure 9A). If a pore diameter is calculated based on the known thickness of the membrane (20 nm) and the conductance of the electrolyte, the initial value is 12.7 nm (explanation in the Supporting Information). While all pores were still clearly conducting an ion current after APS-SMCC-PEG modification, the conductance typically dropped by 90% after in situ modification and 80% after ex situ modification. The small but significant (after analyzing 10 pores) difference can be attributed to the in situ method giving a slightly thicker APS-SMCC layer, which is too dense to allow ions to pass. The 2 kg/mol PEG chains are small enough to assemble quite densely on the surface (0.51 nm^–2^) but the brush does maintain a degree of hydration (at least 80% on a planar surface as shown above). The pore geometry may contribute to a slightly increased volume fraction of polymer when the radius is smaller than the expected brush extension.^68^ Considering all these factors together, the conductance changes seem very reasonable for pores with walls coated by a few nm of compact APS-SMCC and the rest of the volume containing hydrated PEG. For comparison, the 20 kg/mol PEG did not cause an equally large reduction in conductance (data not shown). This is most likely because of a strongly reduced grafting density inside the pore since the 20 kg/mol PEG chains are so large that the negative surface curvature plays a role for the pores we used (diameters in the range 10–20 nm).

As far as we are aware, the results in Figure 9 represent the first example of pure PEG brush formation on standard nanopores in silicon nitride. Previous work by Awasthi et al.^69^ showed how various block copolymers could be used to reduce clogging of nanopores, but the performance was not better than lipid bilayers and the noise in the ion current trace (which must be kept low to detect single molecules) tended to increase. Our PEG brushes instead reduced the noise level (Figure 9B), at least in the low-frequency region (<1 kHz). This may be because each chain is covalently attached, making it unable to move laterally on the surface. Also, after the sulfo-SMCC step all surface charges are removed, which is expected to reduce noise.^70^ Hence, because of the excellent antifouling ability demonstrated in this work, our method can eliminate the problem of clogging while also simplifying detection of protein translocation.^15^ Our modification protocol will also be of interest for creating more advanced polymer-modified nanopores aiming for new applications.^9^

## Conclusions

We have presented a new method for passivation and/or biofunctionalization of silica surfaces with many important advantages compared to existing protocols. First, the method is easy to perform in comparison with, for instance, surface-initiated polymerization, as it only requires exposing the surface to three commercially available chemicals that can be kept in stock. Second, the modified surfaces have superior antifouling performance (98–100% reduced adsorption of serum biomolecules) in comparison with other grafting-to approaches and are clearly competitive with grafting-from methods. Third, further functionalization via biotin is feasible for extremely selective biomolecule capture. Fourth, our method is compatible with silica nanostructures such as nanochannels and nanopores, demonstrating broad applicability for analytical purposes. For the case of nanopores, the noise in the ion current is lower after the surface modification. The ex situ version of our method has been optimized for creating monolayers of APS and sulfo-SMCC, but requires that the sample is rinsed and dried between the steps and mildly heated at one point. Alternatively, the in situ method can be used, where three serial injections are performed, which yields equally well performing surfaces in terms of antifouling, but with a total thickness that is slightly higher on average and has more variation.

Finally, we point out that our method does have some limitations. First, it is not suitable for providing long-term antifouling properties due to the susceptibility of PEG to oxidation in ambient conditions. However, the aim of this work was to develop a method that can be easily used for experimental research with silica surfaces and nanostructures. Also, our method is strictly speaking not “one step” as three chemicals need to be introduced in series. Yet the whole process is easy to perform in less than 1 h, requires no specific expertise, and is compatible with automation. Hence, we believe that this method will become widely used in nanobiotechnology.

## Supporting Information Available

Experimental, NMR spectra, additional ex situ data, APS binding in water, data for APTES, additional XPS data, SPR modeling, control experiment without APS, additional results for gold surfaces, additional in situ data, results for dithiol-PEG, contact angles, PLL-g-PEG results, fouling on commercial sensor slides, additional results on nanochannels, and nanopore formation (PDF).
