# Supplementary material for: Polymer Brushes on Silica Nanostructures Prepared by Aminopropylsilatrane Click Chemistry: Superior Antifouling and Biofunctionality
Source: ACS Appl Mater Interfaces. 2023 Feb 11;15(7):10228–39. doi: 10.1021/acsami.2c21168 (PMC9951205; doi:10.1021/acsami.2c21168)
Supplement: Supplementary file 1 — am2c21168_si_001.pdf [file am2c21168_si_001.pdf]

Polymer Brushes on Silica Nanostructures Prepared by Aminopropylsilatrane Click Chemistry: Superior Antifouling and Biofunctionality

*John Andersson,[1] Julia Järlebark,[1] Sriram KK,[2] Andreas Schaefer,[1] Rebekah Hailes,[1] Chonnipa Palasingh,[1] Bagus Santoso,[1] Van-Truc Vu,[3] Chun-Jun Huang,[3†] Fredrik Westerlund[2] and Andreas Dahlin.[1]**

1 Department of Chemistry and Chemical Engineering, Chalmers University of Technology, 41296 Gothenburg, Sweden.

2 Department of Biology and Biological Engineering, Chalmers University of Technology, 41296 Gothenburg, Sweden.

3 Department of Chemical and Materials Engineering, National Central University, Taoyuan 32023, Taiwan.

† Additional affiliations: (i) R&D Center for Membrane Technology, Chung Yuan Christian University, Taoyuan 32023, Taiwan. (ii) NCU-Covestro Research Center, National Central University, Jhong-Li, Taoyuan 32023, Taiwan.

* Corresponding author: adahlin@chalmers.se

Experimental

Chemicals: ASTM research grade Type 1 ultrafiltered water (milliQ, 18.2 MΩcm) was used for diluting all aqueous solutions. Ethanol (99.5 % or 95 %) was purchased from Solveco. H_2O_2 (35 %) was purchased from Scharlau. Acetone, n-hexane (95+ %), sulfosuccinimidyl 4-(N-maleimidomethyl)cyclohexane-1-carboxylate (sulfo-SMCC), 1-ethyl-3-(3-dimethylaminopropyl)carbodiimide hydrochloride (EDC, Premium-grade), and avidin conjugated with fluorescein (FITC) were purchased from Thermo Fischer Scientific. NH_4OH (25 %) was purchased from Acros Organics. Thiol-PEG-methyl-ether 20 kg/mol (PDI 1.01) and 2 (1.8) kg/mol and biotin-PEG-SH 2 (2.2) kg/mol were purchased from Laysan Bio Inc. Isopropanol (2-propanol, ≥ 99.4 %), bovine serum (adult), bovine serum albumin (BSA, ≥ 98 %), avidin (from egg white, ≥ 98 %), (3-aminopropyl)trimethoxysilane (APTES, 99 %), poly(ethylene glycol) dimethyl ether (non-thiolated PEG, 2 kg/mol), sodium dodecylbenzenesulfonate (SDS), potassium chloride (KCl, ≥ 99 %) and sodium sulphate (Na_2SO_4, ≥ 99 %) were purchased from Sigma Aldrich. Phosphate buffered saline tablets (PBS, 0.01 M Na_2HPO_4, 0.0027 M KCl and 0.137 M NaCl), poly(ethylene glycol) dithiol (HS-PEG-SH, M_n = 1.5 kg/mol), poly(ethylene glycol) dimethyl ether (mPEGm, M_n = 2 kg/mol), N-hydroxysulfosuccinimide sodium salt (NHS, > 98%), 4-morpholineethanesulfonic acid (MES, > 99.5%), methanol (ACS reagent, ≥99.8%), triethanolamine (>98 %), toluene (anhydrous, 99.8%), NaOH (reagent grade, ≥98%,), TCEP and ethanolamine (> 99 %) were purchased from Sigma Aldrich. PLL-g-PEG with 20 kg/mol PLL and one 2 kg/mol PEG chain grafted at every 3.5 monomer on average were from SuSoS Surface Technology. Buffers were degassed before used.

Surfaces: Borosilicate cover glass slides were purchased from VWR. QCMD sensors with SiO_2 coatings (< 1 nm RMS roughness) were purchased from Biolin scientific. SPR sensors with a SiO_2 coating were prepared from glass substrates (Bionavis) according to the following procedure: regeneration using RCA2 cleaning (HCl : H_2O_2 : water at 1:1:5 volume ratios at 80 °C for 30 min), O_2 plasma treatment at 50 W, 250 mTorr, 80 sccm for 60 s and metal deposition of 2 nm Cr and 50 nm Au using electron beam physical vapor deposition (Lesker PVD 225). Deposition of SiO_2 was performed immediately after metal deposition and an additional 60 s O_2 plasma treatment using ALD (Oxford FlexAL) at 300 °C with a bis(t-butylamino)silane precursor and O_2 as process gas. Carboxymethyl dextran (3D gel matrix) coated chips (standard density) were purchased from Bionavis.

Silatrane synthesis: The synthesis of aminopropylsilatrane (APS, Scheme S1) was performed as previously reported,[S1] with slight modifications. APTES (2.2 mL, 10.5 mmol), triethanolamine (0.8 mL, 6 mmol), MeOH (7 mL), toluene (15 mL) and NaOH (55 mg, 1.5 mmol) was refluxed at 85 ˚C for 24 hrs. The solvent was removed by rotary evaporator and the slurry was poured into a stirred solution of *n*-hexane (50 mL). The white solid was then collected by vacuum filtration and dried in vacuum overnight. 1.3 g of APS was obtained as a white solid (91 % yield). The product was then immediately dissolved in DI water (460 mM) as a stock solution and stored at 4 ˚C. ^{1}H NMR (400 MHz, CDCl3) δ: 3.75 (t, J = 5.8 Hz, 6H, Hd), 2.79 (t, J = 5.8 Hz, 6H, He), 2.61 (t, J = 6.9 Hz, 2H, Ha), 1.55 – 1.44 (m, 2H, Hb), 0.47 – 0.30 (m, 2H, Hc).

Sample cleaning: SPR and QCMD sensor surfaces were first cleaned by rinsing with a water stream, followed by ultrasonication in acetone for 5 min, sonication in isopropanol for 5 min, drying using a gas stream of N_2 and finally UV O_3 treatment for at least 20 min (Compact UV-Ozone Cleaner from Cyky). An additional immersion in 99.5 % EtOH with N_2 drying was performed for plain gold SPR sensors to reduce Au-OH groups after UV O_3 treatment.[S2] Nanochannels were cleaned with 0.2 µm syringe filtered 1 % (v/v) Hellmanex. Membranes for nanopore formation were cleaned with piranha (conc. H_2SO_4 and 30 % H_2O_2 mixed in volume ratio 3:1 for 20 min).

Silanization: The methodology is described throughout the text and in Figure 1. For the *ex situ* method, a droplet of APS (115 µM in 99.5 % EtOH) was placed on the surface for 1 min. For the *in situ* method, the solution was injected into the liquid cells and the incubation time was longer (~5 min). When functionalizing nanostructures, additional rinsing in 95% ethanol was done after binding.

Crosslinker binding: For the *ex situ* method, sulfo-SMCC at 0.5 g/L in 10× diluted PBS buffer was placed on the sample for 1 min. For the *in situ* method, the solution was introduced by steady flow using concentrations in the range 0.5-2 g/L and the incubation time was longer (~15 min).

Polymer grafting: The protocol was similar to that previously used for modifying gold.[S3] For *ex situ* modification, the silica modified with APS and sulfo-SMCC was placed inside a petri dish containing 2 or 20 kg/mol thiol-PEG dissolved at 1 g/L or 0.12 g/L in 0.9 M Na_2SO_4 and incubated for 1 or 2 h respectively on a shaker table set to 100 rpm at room temperature. A liquid stream of water was used to rinse the sample, followed by drying in a stream of N_2. For *in situ* modification, the same PEG solutions were injected into the different liquid cells.

AFM measurements: A NTEGRA AFM (NT-MDT) instrument was used with Tap300Al-G tips (BudgetSensors®) with 200-400 kHz resonant frequency, 20-75 N/m force constant and 10 nm tip radius. The tapping mode AFM images were recorded. Post-processing of data includes levelling using a mean plane subtraction, followed by row alignment using a second order polynomial and offsetting to zero at the lowest measured height.

XPS measurements: Borosilicate cover glass slides were used as substrates. The APS sample was prepared by incubating in 460 μM APS in 99.5 % EtOH for 5 min, followed by rinsing in 95 % EtOH (no curing) and immersion in 10× diluted PBS for 5 min before a final water rinse and N_2 drying step. The same procedure was followed for the sample with APS and sulfo-SMCC with incubation in 10× diluted PBS at 1 g/L for 30 min. The XPS measurements were conducted at a PHI 5000 VersaProbe III Scanning XPS Microprobe instrument employing a monochromated Al K_α X-ray source (1486.7 eV). The spectra were measured with a beam size of 200 μm^2 at 50 W and 15 keV. Carbon 1s spectra were measured first. Charging of the samples was compensated by an electron gun and an Ar^+ ion source. The binding energy was calibrated for each sample afterwards by shifting the binding energy range such that the leading C 1s peak appears at 285 eV, as in previous studies of similar films.[S4-S7] The intensity of the spectra was normalized to the low binding energy side of the background. The spectra were deconvoluted by Voigt profiles for which the Lorentzian and Gaussian widths are individual parameters. The Lorentzian width was fixed to a literature value for the specific core hole.[S8] Prior to fitting a Shirley type background was subtracted.

SPR measurements: A Bionavis 220A Navi multiparameter SPR instrument was used. The diode wavelength was 670 nm. For dry films, the reflectivity was measured with a single scan between 39.2° and 50.0° (duration 2.19 s), while continuous scanning between 58.0° and 77.9° (duration 3.88 s) was used in liquid. Layer thickness for dried films as well as exclusion heights were determined with non-linear least-square fitted Fresnel models implemented with the transfer-matrix method using custom MATLAB code.[S9-S11] Each SPR sensor was measured prior to further functionalisation to obtain an individual Fresnel model background, thus accounting for any initial SiO_2 thickness variations between different SPR sensors. All liquid injections were performed at a flow rate of 20 μL/min with a temperature set to 25 °C.

QCMD measurements: A Q-Sense E4 instrument (Biolin Scientific) was used for the QCMD measurements, and a NE-1000 syringe pump (New Era pump systems) together with an Idex V-

451 manual injection valve (Genetec) were used for flow control and purging air bubbles. The temperature was set to 25 °C and the flow rate was 150 µL/min. Data analysis and modelling was performed with Qtools, as described previously[9] using overtones 3, 5, 7, 9 and 11. The density of the film was assumed to be 1000 kg/m^3. Overtone three was excluded in some cases when modelling the viscoelastic thickness with the Voigt model[S12] to acquire a better overall fit. The shear modulus was assumed to be independent of frequency.

Fluorescence microscopy of nanochannels: Further details and fabrication procedures for the nanofluidic devices used in this work is described elsewhere.[S13] Briefly, the device contains micro- and nanochannels made in silicon dioxide that are sealed with borosilicate glass (Si-Mat, Germany) using high-temperature bonding. Liquid was injected by loading 15 µL in reservoirs and applying 400 mbar with N_2. An inverted microscope (Zeiss AxioObserver.Z1) equipped with a 63× oil immersion objective, EMCCD camera (Photometrics Evolve) and a 475 nm LED light source (Colibri 7, Zeiss) were used for imaging. Images (512×512 pixels) were recorded at 126 gain and 100 ms exposure time. Flow was pressure driven.

Ion current recording on nanopores: Silicon nitride membranes (40 × 40 µm^2) were prepared in 1 cm^2 Si chips following standard protocols.[S14-S16] Controlled dielectric breakdown[S17] was used to form a pore in a 1 M KCl electrolyte using a commercial system (Spark-E2, Northern Nanopore Instruments). The pores were "conditioned" (grown in size) in 3.6 M LiCl. Conductance was measured by an Axopatch 200B (Molecular Devices) in 1 M KCl. To record the current baseline, a steady potential of 0.1 V was applied at sampling rate 25 kHz and bandwidth of 100 kHz.

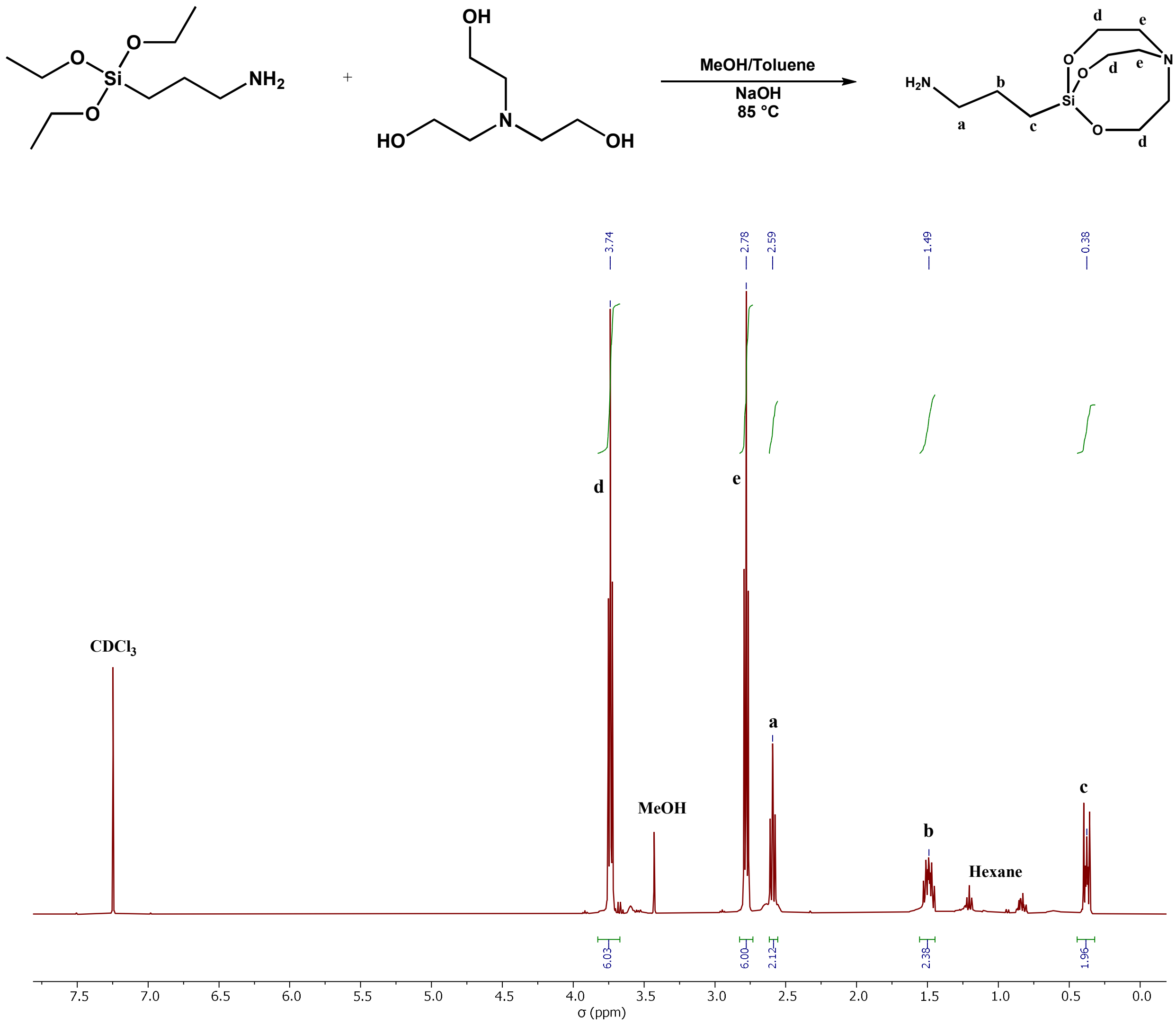

Scheme S1 Synthesis of APS; ^{1}H NMR spectra shown.

Non-monolayer formation of APS and SMCC (*ex situ* data)

The *ex situ* method was optimized for creating monolayers of both APS and sulfo-SMCC with the specified concentrations, solvents and incubation times. For the *in situ* method, one needs to be somewhat careful with concentrations and incubations times to get as close to monolayers as possible, and introduce sulfo-SMCC reasonably fast after the APS. In this section we show some data on what happens if the protocols are not followed. The main purpose is to give an idea of how precisely they need to be obeyed. As will be shown, there is quite some tolerance with respect to most factors. These supplementary results can also be useful for further understanding the chemical reactions on the surface.

We first consider the APS and sulfo-SMCC binding. Using higher concentrations and longer incubation times generally resulted in thicker films (more than a monolayer). Figure S1 shows examples of dry layer thickness determined by SPR. The final PEG layer thickness is also included, but concentration and incubation time were not altered for this step. Although the data set does not present a detailed investigation on effects from concentration and incubation time, it is clear that thicker layers of APS and sulfo-SMCC are generally formed when these parameters are increased. Additionally, the experimental variation is much higher for all layers (including PEG) compared to when the protocol is followed (in which case the variation is ± 0.1 nm). We also noted that while most of the PEG brushes were still antifouling, some of the samples that were not prepared according to protocol did not exhibit this feature.

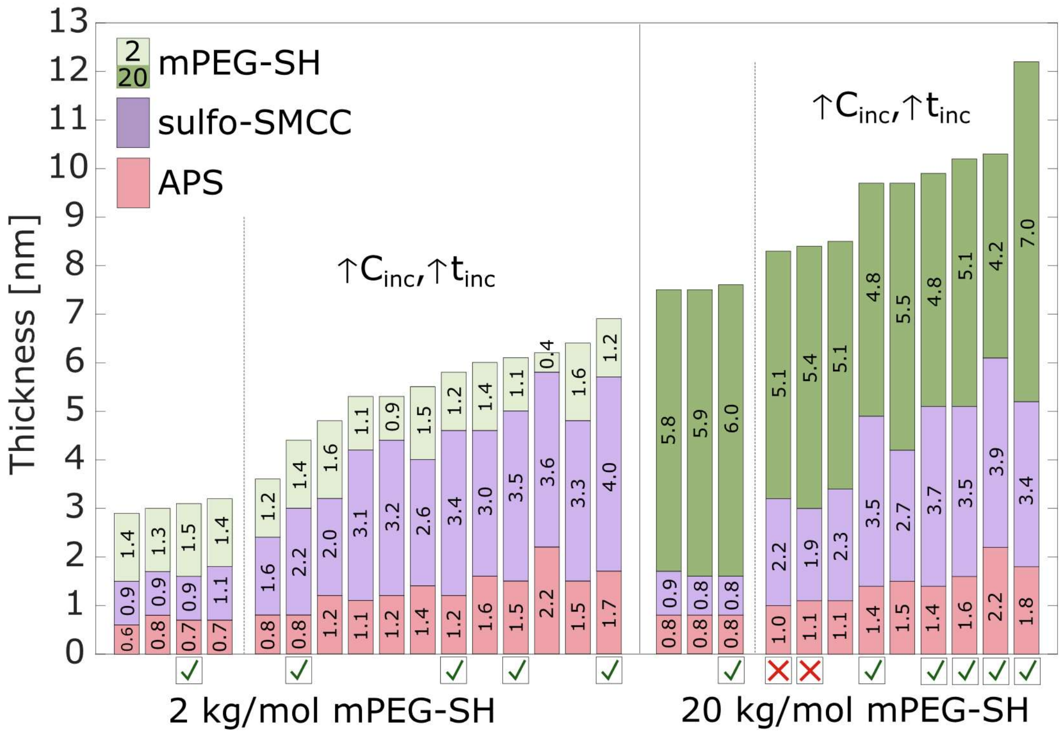

Figure S1 Measured dry thickness of each layer when the concentration and/or incubation time of APS and sulfo-SMCC has been increased. For each molecular weight of PEG, the samples to the left of the dashed line were prepared according to the *ex situ* protocol, while the concentration and/or incubation time was increased for the others. Green check box means that the PEG brush fully repelled BSA (no binding detected), red cross means that it did not.

The effect of incubation time for APS in ethanol (99.4%) or water at different concentrations was further investigated (Figure S2). At 460 µM in ethanol there is a tendency of forming multilayers already after ~1 min. We believe this is because APS precipitates on the surface since it is close to its solubility limit at these conditions, as supported by other observations. For instance, we noted that APS dissolved in ethanol at 460 µM over time produced a turbid solution if stored at 4 °C. In comparison, lowering the concentration to 115 µM (i.e. according to protocol) gave monolayer thickness (Figure S2). Furthermore, incubation in water gives less than a monolayer on the surface even after 1 h, showing the importance of having the right solvent.

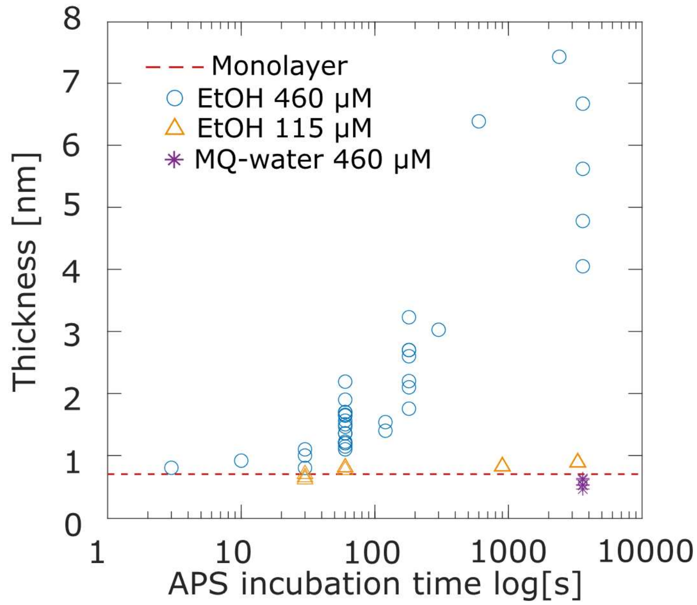

Figure S2 Multilayer formation of APS. Thickness of the final cured APS layer thickness after a given incubation time in 99.4 % ethanol or water at the specified concentration. Each mark corresponds to a different sample surface. All surfaces were rinsed in a stream of 95 % ethanol after incubation, cured and rinsed in a stream of water. The red dashed line corresponds to a monolayer of APS at 0.7 nm.

One reason why sulfo-SMCC can give multilayers at increased concentrations may be the formation of micellar structures. Figure S3 shows the surface tension as a function of total sulfo-SMCC concentration, suggesting amphiphilic properties. We could, however, not investigate micelle formation in this manner due to the solubility limit of sulfo-SMCC at 5 g/L. A OneAttension Theta goniometer from Biolin Scientific was used to measure surface tension using pendant drop method. Surface tension was recorded at 15 fps for 10 s (values are time-averaged). Before starting measurements, the glass syringe and needle were cleaned in 1% SDS and water. The surface tension of a reference droplet of water in room temperature was found to be 72.6-72.7 mN/m. All sample surfaces were dried with a gas stream of N_2 immediately prior to contact angle measurements. Measurements were performed in the order of low to high concentrations.

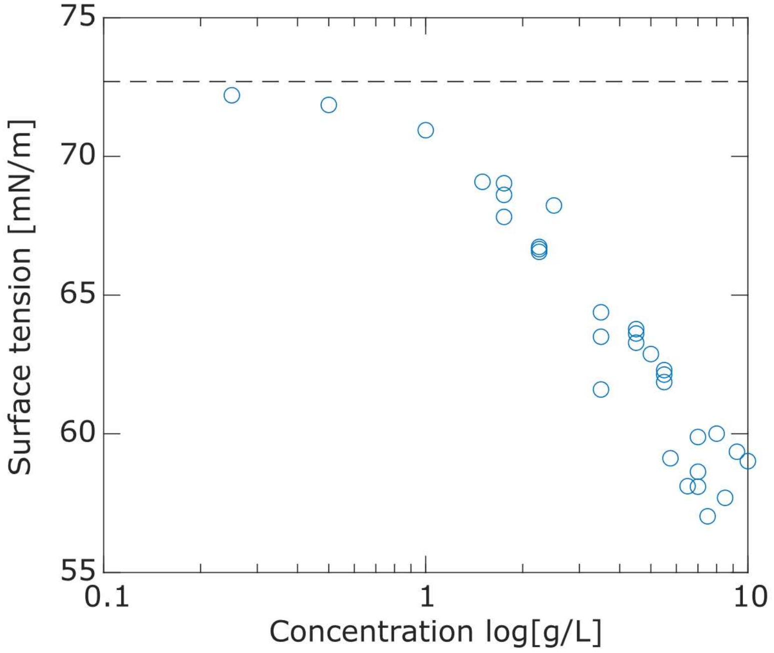

Figure S3 Surface tension of solutions of sulfo-SMCC in 10× diluted PBS.

Besides forming more than a monolayer, the APS layer can also slowly hydrolyse to sub-monolayer coverage unless cured and/or protected by sulfo-SMCC. Figure S4 shows how the APS thickness decreases when incubating different samples in water. Interestingly, already for the initial thickness we noted an influence from the solvent type used to briefly rinse the sample after APS incubation. The lower the polarity of the rinsing solvent, the more APS remains, indicating an adsorption mechanism via hydrogen-bonding or charge interactions that may be overcome by a polar solvent. If water is used for the rinsing step, the initial layer thickness is already much below monolayer (Figure S4). A similarly strong desorption effect in water has been observed previously for APTES.[S18] The curing improves the stability of the layer, in line with previous observations for silanes[S18] and can likely be inferred from the increased rate of siloxane bond formation induced by heat, replacing the initial non-covalent adsorption.[S19-S21]

Note that as explained in the main text, curing is convenient but not a critical step because the subsequent sulfo-SMCC layer can also be used to stabilize the initial APS layer towards hydrolysis.

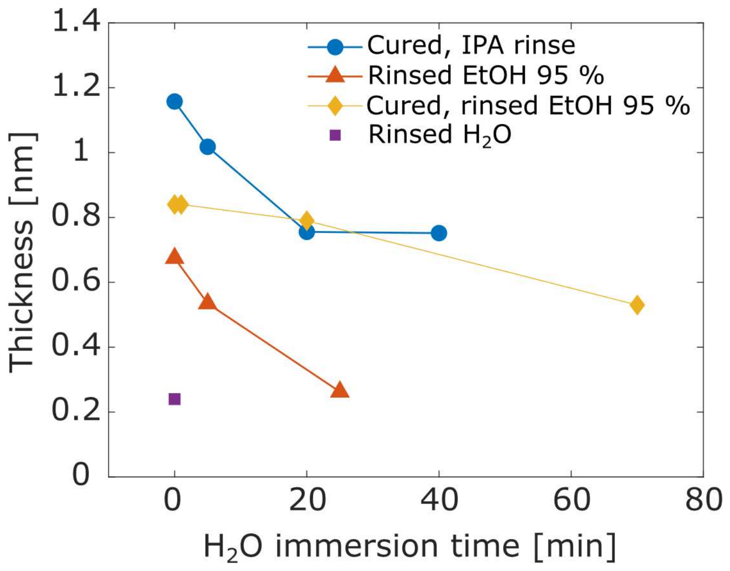

Figure S4 Stability towards hydrolysis. The thickness of APS on SiO_2 was measured in SPR for different treatments after APS incubation (performed according to the *ex situ* protocol). The samples were rinsed for ~10 s with a stream of different solvents after which some were cured. All measurements were performed after these steps. (Note that one sample was rinsed with water but not immersed in water.) Each trace corresponds to one sample surface. Note that even a quick rinse in water desorbs much of the APS if the sample has not been cured.

Additional data on *in situ* modification using water

To confirm the importance of the solvent for the APS binding, we also tested to use water during *in situ* modifications monitored with SPR (Figure S5). Although the signal from APS seems to saturate at a value which is close to the expected signal from a monolayer, much is quickly desorbed as soon as the surface is rinsed with water again, in agreement with the data in Figure S4. This shows that water is not suitable as solvent for promoting APS binding to silica.

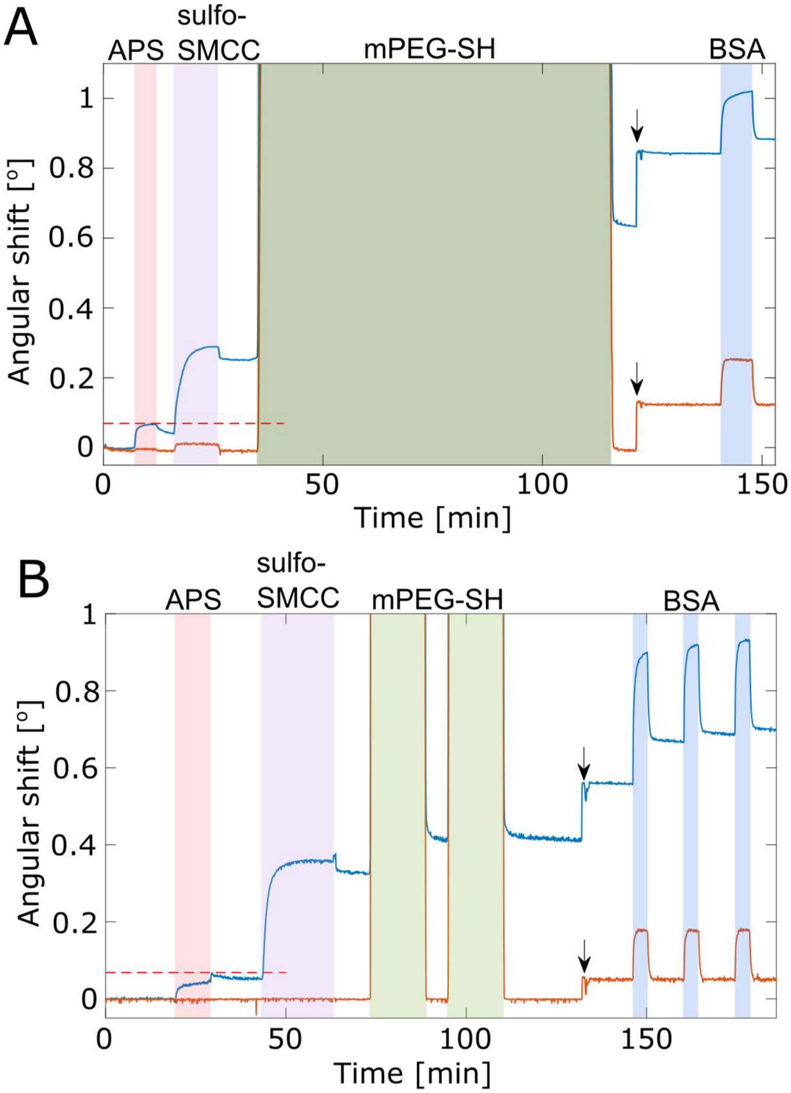

Figure S5 *In situ* modifications in SPR with water as solvent for the initial APS binding. The signal from APS is lower and the signal from sulfo-SMCC is higher. PEG binding works but the resulting surfaces are not antifouling (BSA in PBS adsorbs considerably). The arrows show the change to PBS as running buffer.

Data for APTES modification

We managed to prepare APTES layers with an average thickness very similar to APS. An example of SPR results is shown in Figure S6, where the dry thickness was determined to 0.74 nm. However, the APTES films were still more rough (see main text) and vapor phase deposition was required for reproducibility. This is especially problematic (if possible at all) when modifying, for instance, the interior of nanochannels.

Vacuum deposition of APTES was performed inside a sealed glass bell chamber connected to a DIVAC 1.4HV3C vacuum pump. While containing the cleaned silica sample, the chamber was primed by a twice repeated pump down cycle to reduce the chamber humidity and moisture content. Subsequently, a droplet of APTES was placed on a clean microscope objective glass next to the sample, following immediate evacuation to low pressure (order of a few mbar) after which the chamber was sealed from the pump with a valve and the droplet was left to evaporate for 40 min. Finally, the sample was taken out of the chamber and cured like the APS layer.

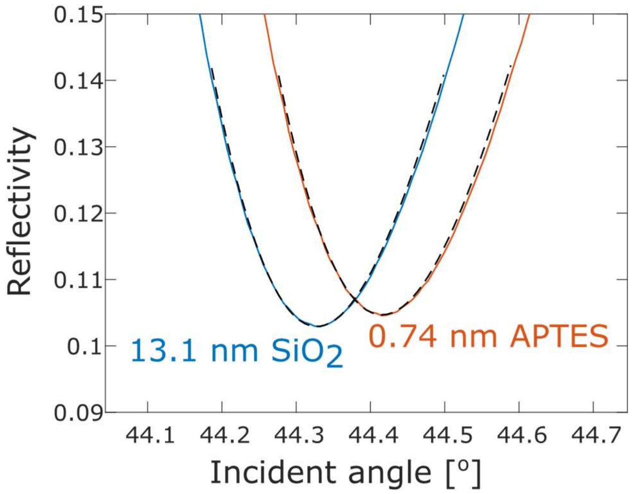

Figure S6 SPR spectra before and after APTES vapor-phase functionalization. The thickness of the film is the same as obtained for APS (assuming the same RI).

Additional XPS analysis

To confirm the click chemistry reaction between the primary amine in APS and the sulfonated NHS group in sulfo-SMCC, we performed XPS as described in the main text. Additionally, we also looked at the S 2p region to confirm that there was only trace amounts of sulphate left on the surface[S22] (Figure S7A). This confirms that the crosslinker is binding in the expected manner to the amine groups on the surface. The survey spectrum is shown in Figure S7B.

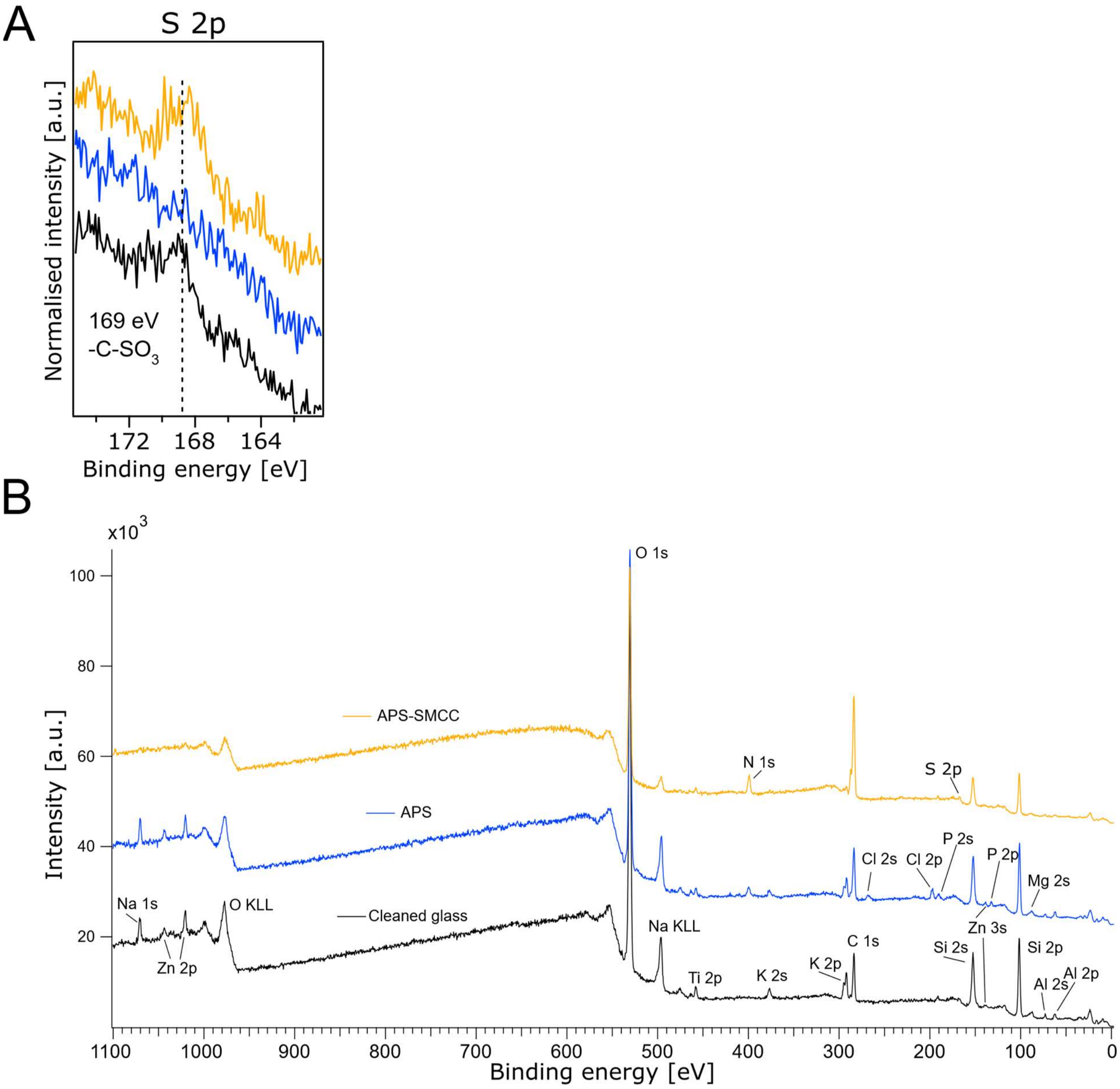

Figure S7 (A) XPS spectra of the sulphate peak region for glass (black), after APS (blue) and after sulfo-SMCC (yellow). After sulfo-SMCC the S 2p signal is not significantly larger than for the other surfaces. (B) Survey spectrum.

Including the silica coating in SPR spectra modelling

To characterize the silica coating and its influence on the SPR sensor we first performed Fresnel fitting of spectra after ALD deposition (Figure S8). Using a literature value for the (real) refractive index of SiO_2 (Table S1), we fitted both thickness and extinction coefficient of the film, which depend on the SPR minimum angle and resonance width, respectively. In this step, values previously fitted for Cr and Au for bare sensors[S10] were used. Each organic layer was assumed to have a certain (purely real) refractive index and a thickness was fitted subsequently after each step. All values are summarized in Table S1.

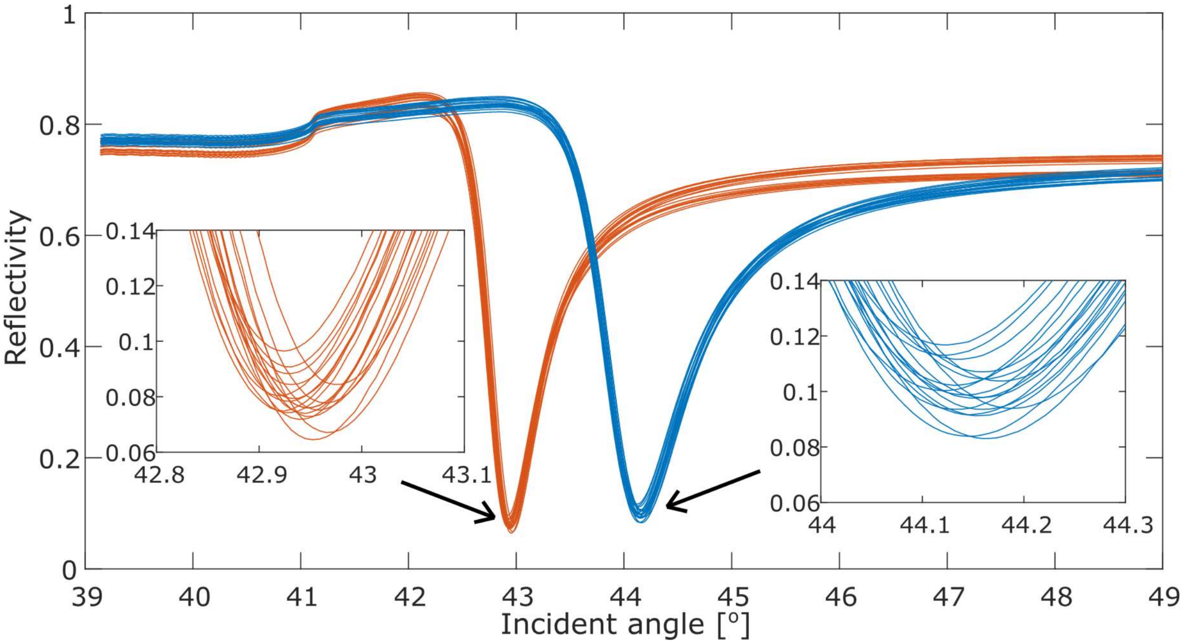

Figure S8 SPR spectra of bare Au sensors and after SiO_2 coating (10 nm by ALD). 18 different samples are included to illustrate variation.

For quantification of adsorbed amounts in liquid, the bulk sensitivity (S_0) and field decay length (δ) are needed. While these parameters are well known on bare Au sensor surfaces,[S3] they may be significantly influenced by the thin silica coating. To investigate if this is the case we performed Fresnel simulations and solved the dispersion relation for the surface plasmon to calculate the field distribution[S23] (Figure S9). The final values obtained for S_0 and δ were in the end very similar to those for bare gold (within a few percent). The silica coating simply needs to be thicker to have a strong effect. One can thus quite accurately compare signals from silica SPR sensors with those of gold sensors without any correction factor.

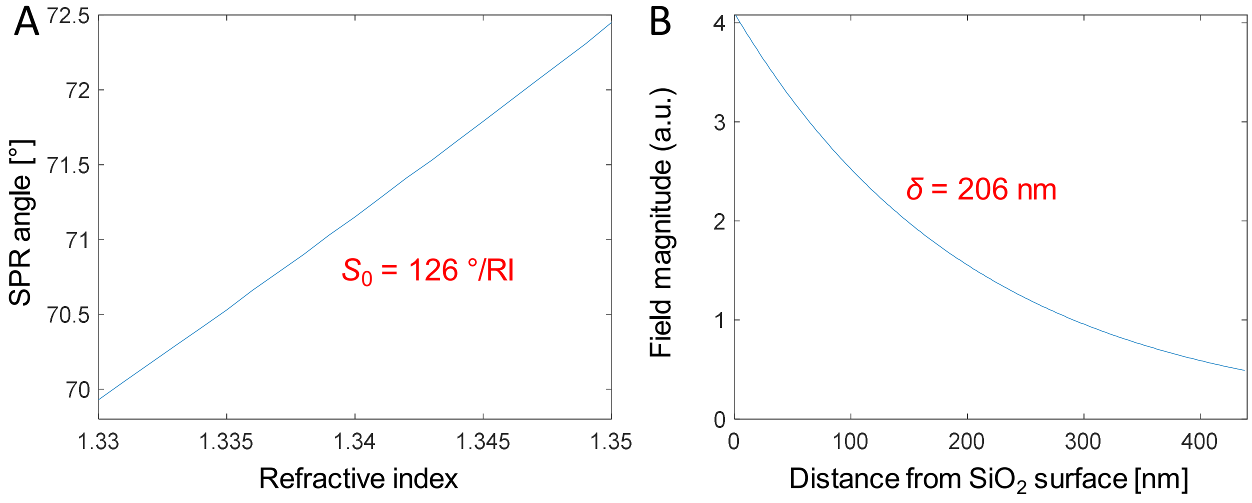

Figure S9 Simulated bulk sensitivity and field extension for silica coated SPR surfaces. (A) The refractive index was increased from that of water in small steps and the angle of minimum reflection was determined for each calculated spectrum. (B) Field plot, where a distance of zero means the SiO_2-water interface (not gold-SiO_2). The values of S_0 and δ were determined from a linear and an exponential fit, respectively.

Layer	*d* (nm)	*n*	*k*
**Prism/Substrate glass**	∞	1.5202	0
**Chromium**	2	3.3105	3.4556
**Gold**	50	0.2238	3.9259
**SiO_2 (ALD)**	Fitted	1.4628	0-0.1 (fitted)
**APTES/APS**	Fitted	1.42	0
**Sulfo-SMCC**	Fitted	1.42	0
**mPEG-SH**	Fitted	1.456	0
**Protein**	Fitted	1.52	0
**Bulk medium**	∞	From TIR angle	0

Table S1 Thickness (*d*), refractive index (*n*) and extinction coefficient (*k*) used in Fresnel models at 670 nm wavelength. Note that the fitting is performed in multiple steps: bare sensor, after SiO_2 and then after each organic layer. The values for the previous layers are kept fixed when each new layer is modelled.

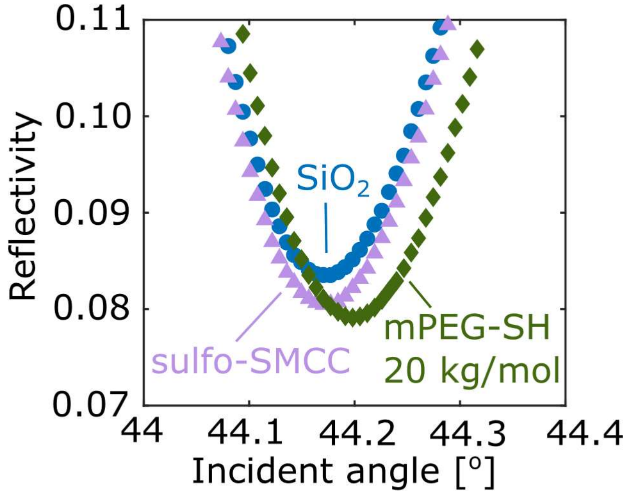

Figure S10 Control experiments showing SPR spectra after exposure to sulfo-SMCC and PEG without the initial APS modification. The spectral changes are very small (compare with results in main text).

Physisorption of disulphides on gold

While the current work is about the development of a method for modifying silica, we consider it informative to compare with the results obtained when grafting the same PEG chains to gold as we have done in previous work.[S3, S9] One observation from those studies is that the grafting density, as determined from the dry thickness after grafting, varied depending on batch and supplier of PEG even if the specified molecular weights were the same. (For instance, for 20 kg/mol grafting densities in the range 0.2-0.3 nm^{-2} have been obtained.)

We confirmed that PEG without thiols did not physisorb to gold (Figure S11A). Also, we noted that upon immersing samples with high PEG amounts, corresponding to grafting densities in the higher range (close to 0.3 nm^{-2} for 20 kg/mol) in water, there was always a downward drift in the baseline in SPR. We left the samples immersed in water for different amounts of time and measured how the dry thickness changed (Figure S11B). Indeed, after sufficient time the PEG amount on the surface started to stabilize on values that were corresponding to grafting densities in the lower range (~0.2 nm^{-2} for 20 kg/mol). This shows that many chains are physisorbed, not properly end-grafted (and the "true" grafting density on gold for 20 kg/mol PEG is thus 0.2 rather than 0.3 nm^{-2}). We hypothesized that the physisorbed molecules may be disulfides and indeed, introduction of TCEP eliminated the physisorption on gold (see main text).

We see two reasons why additional "dimer" PEG chains (linked by disulfides) could physisorb on the gold surface, even when it contains a PEG brush formed by the corresponding thiol-PEG "monomer". First, disulfides have a known affinity for gold just like thiols. Second, the dimeric chains will have much higher molecular weight, which means that they precipitate more easily, and the grafting solution is already tuned to be close to the cloud point. Hence, the brush and the solid surface may act as a nucleation site for precipitation of the dimers specifically.

Note that although adding TCEP may be a convenient way to only get properly end-grafted chains on gold, this chemical must not be used when grafting to silica because it interferes with the bond formation with maleimides.[S24] Since the physisorption of disulfides does not occur on silica, this is not an issue in practice. However, care must be taken to ensure that the thiol-PEG used does not contain TCEP as an additive. Also, if using TCEP when grafting to gold, it should be kept in mind that the chemical degrades at high pH.[S25] However, the pH will automatically be low enough (2-3) when TCEP (25 mM) is added to the grafting solution (only Na_2SO_4 in water), since it does not contain buffering species.

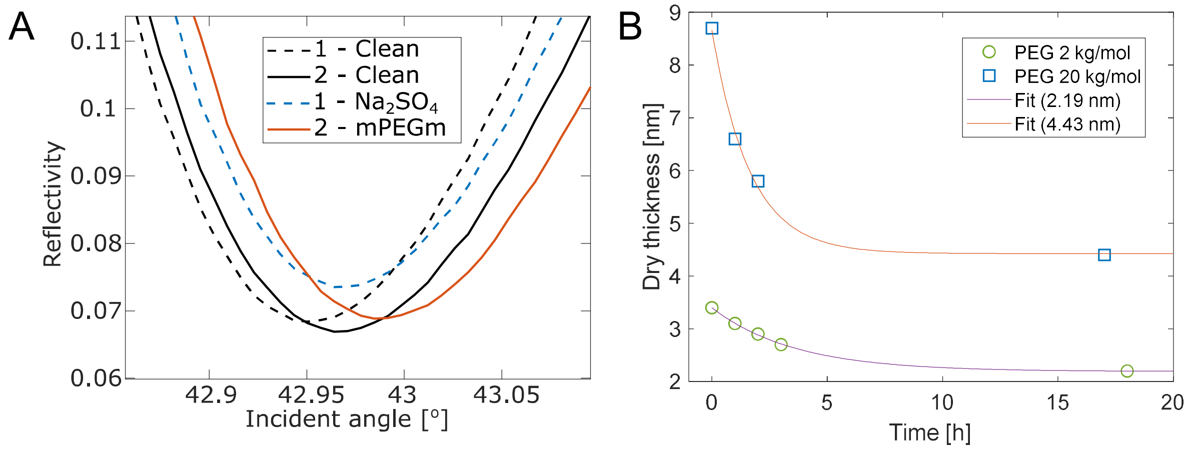

Figure S11 (A) Control experiments showing SPR spectra of gold surfaces (1 and 2) before and after exposure to 0.9 M Na_2SO_4 with or without non-thiolated PEG (1 g/L). The shifts is very small and comparable. (B) Slow spontaneous desorption of PEG from gold surfaces following exponential decays converging to constant values (shown in legend).

Supplementary data for the *in situ* method

Figure S12 shows additional results for the *in situ* modification. In each case, the molecular weight of PEG is that which is not shown in the data in the main text (2 or 20 kg/mol). Overall, the data looks similar and the only noticeable difference is the altered signal from the PEG (it is higher for 20 kg/mol than for 2 kg/mol).

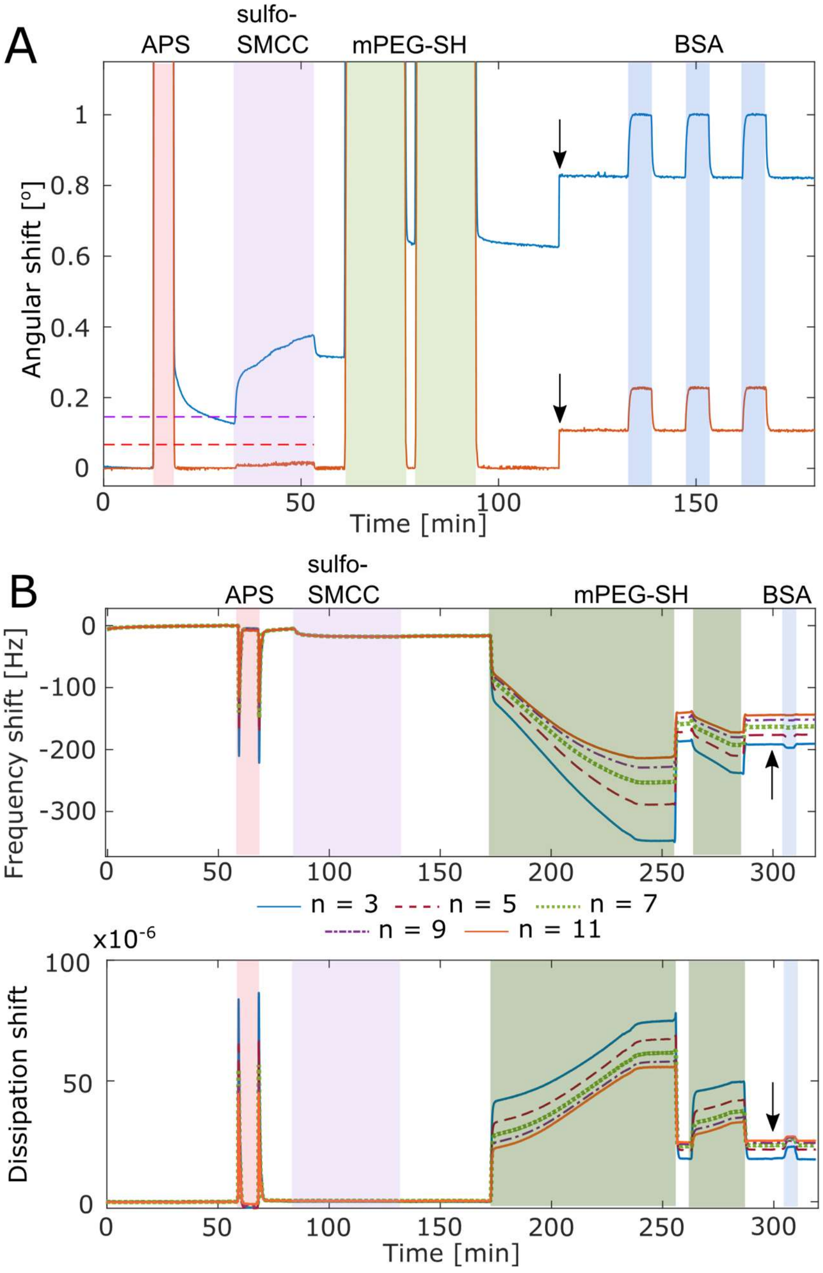

Figure S12 *In situ* modification in (A) SPR and (B) QCMD. This is supplementary data for the other molecular weight of PEG (2 kg/mol for SPR and 20 kg/mol for QCMD).

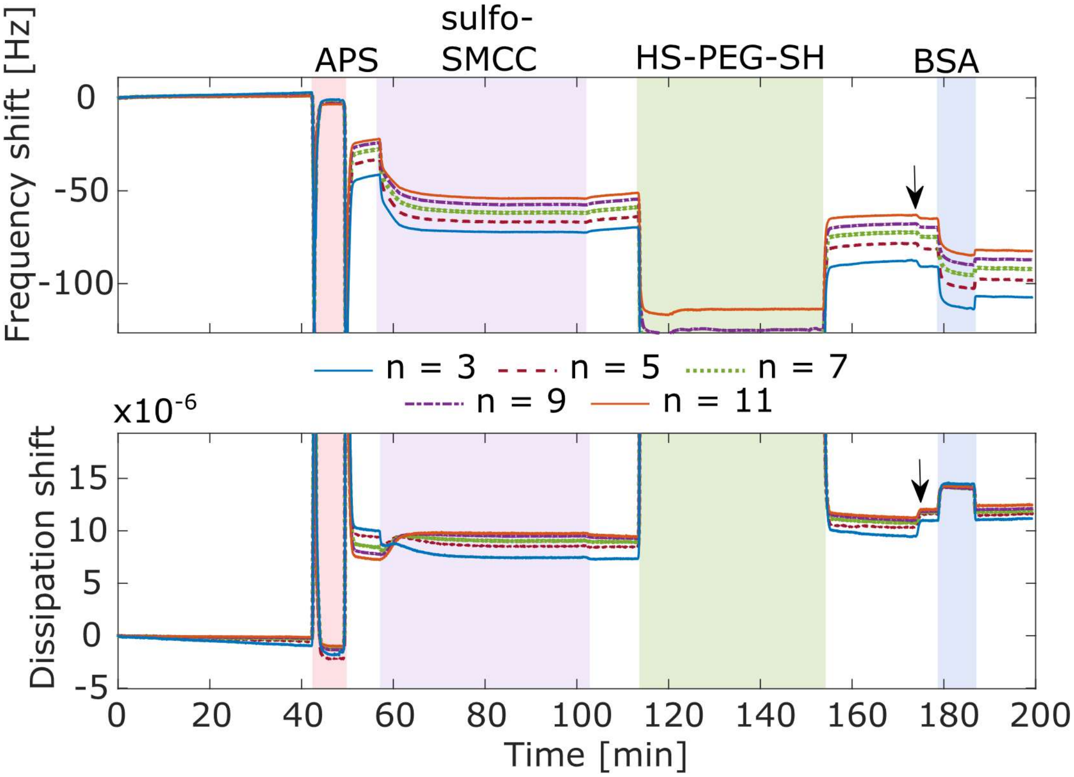

Figure S13 Control experiment showing grafting of dithiol-PEG (1.5 kg/mol, 1 g/L in 0.9 M Na_2SO_4) to SiO_2 in QCMD. The response from the PEG is very low and BSA adsorbs in PBS. Black arrows indicate change from 10× diluted PBS to regular PBS.

Contact angle measurements

While all samples were fairly hydrophilic, the surface became more hydrophobic after the sulfo-SMCC modification (Figure S14). This strengthens the view that this layer protects the APS-SiO_2 bonds from water access (that leads to hydrolysis), which is why the surfaces are stable in water after sulfo-SMCC binding but not before.

A OneAttension Theta goniometer from Biolin Scientific was used to measure static contact angles (sessile drop). Contact angles were recorded 20 s after placing a water droplet on the surface.

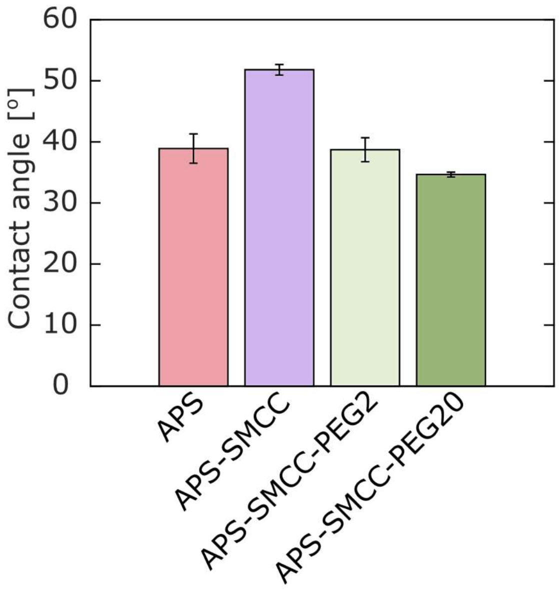

Figure S14 Contact angles measured after the different modification steps.

Quantification of PLL-g-PEG binding and its instability

The surface coverage in mass per area can be estimated by:

$$\Gamma = \frac{d\Delta\theta}{S_0 b\left[1 - \exp\left(-\frac{2d}{\delta}\right)\right]} \quad (S1)$$

Here S_0 is the bulk sensitivity, δ is the decay length of the evanescent field, b is the refractometric constant and $\Delta\theta$ is the shift in the SPR angle. The film thickness is d. Given that $d << \delta$, which is the case for PLL-g-PEG and the APS-SMCC-PEG (at least for 2 kg/mol PEG), the expression can be simplified:[S3]

$$\Gamma = \frac{\Delta\theta\delta}{2S_0 b} \quad (S2)$$

Before using this expression, the parameters S_0 and δ need to be estimated. We use the simulated values from Figure S9: S_0 = 126 degrees per RI unit and δ = 206 nm. This gave the PLL-g-PEG coverage reported in main text based on the signal observed from binding (0.13°, Figure S15A). Protein coverage in liquid state was calculated in the same manner but with the standard refractometric constant of b = 0.182 cm^3/g.

We tested the stability of the PLL-g-PEG layer with respect to the surfactant SDS, which removed around half of the adsorbed amount (Figure S15B). Using a high flow rate further removed more of the block copolymer and the surface was not protein repelling afterwards. This illustrates limitations with non-covalent grafting methods. For comparison, the APS-SMCC-PEG layers were stable when tested in the same manner.

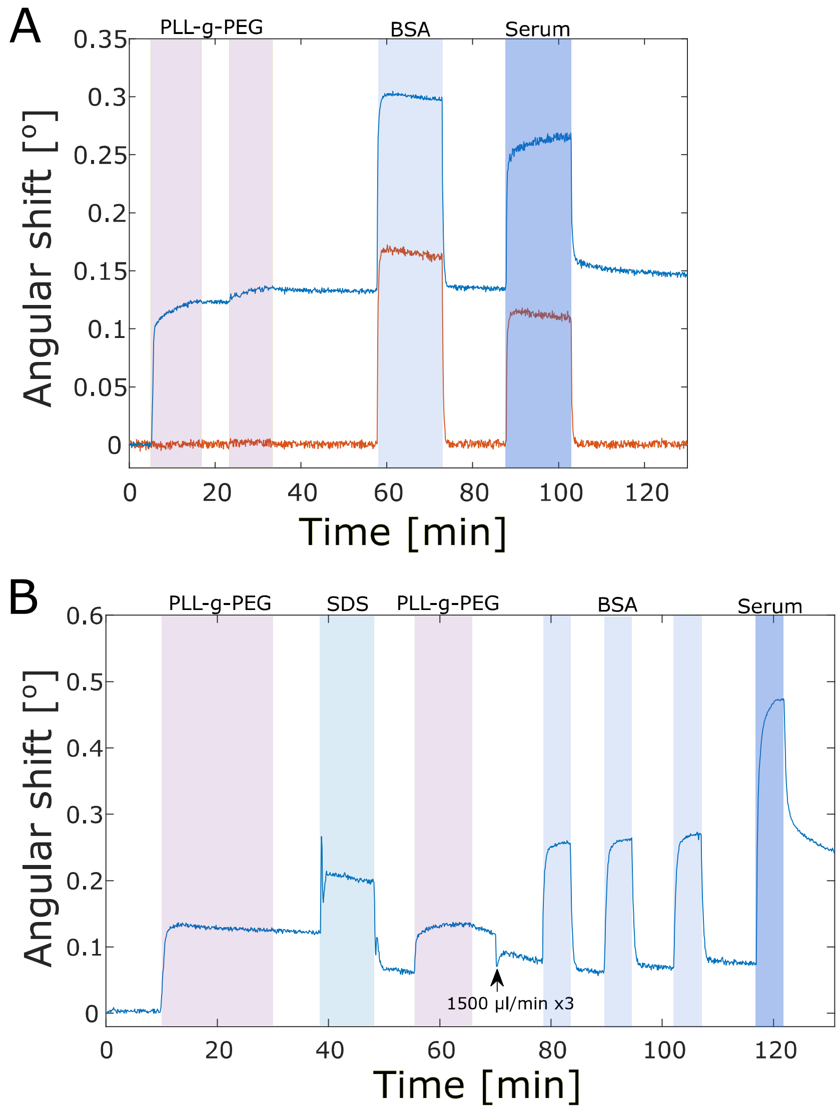

Figure S15 SPR data of PLL-g-PEG. (A) Binding of PLL-g-PEG and exposure to BSA and serum. (B) Testing stability of the PLL-g-PEG with respect to 1% SDS and high flow rate. Afterwards, the surface is exposed to proteins.

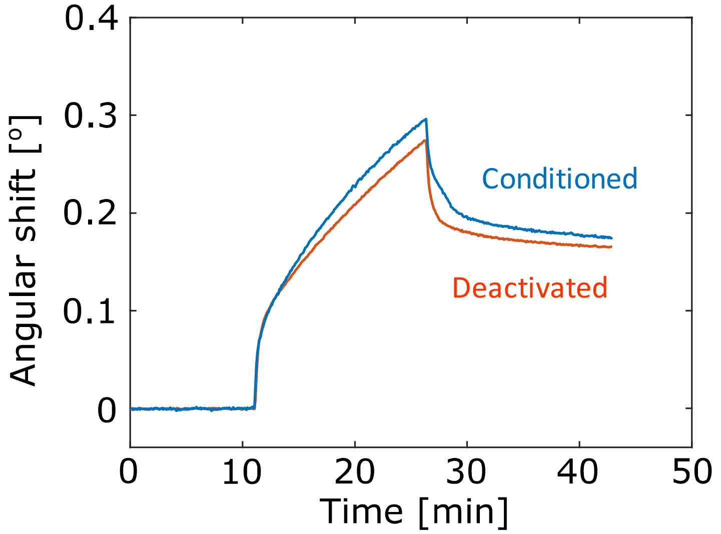

Figure S16 Testing fouling on commercial SPR chips with carboxymethyl-dextran coating. The sensorgrams show injection of 10× diluted serum in PBS and subsequent rinsing. Sensor surfaces were conditioned by injecting 2 M NaCl for 10 min, followed by 10 mM NaOH for 10 min. On one surface, a deactivation protocol with ethanolamine was used to remove carboxyl groups. The protocol (available in the Handbook of MP-SPR from Bionavis) consists of: 50 mM sulfo-NHS and 200 mM EDC in 10 mM MES buffer injected for 10 min, followed by 1 M ethanolamine at pH 8.15 for 10 min. The amount of protein adsorption is considerable on both surfaces.

Intensity in nanochannels during injections

For the nanostructures, an additional rinsing step with ethanol was performed to fully wash out APS from the system before introducing sulfo-SMCC. To ensure that this did not influence the APS, we checked in real-time with SPR if there was any significant desorption when washing with ethanol. Figure S17 shows that this is not the case when tested with 95% ethanol. Assuming that the other 5% is water, the results show that such small amounts of water do not lead to any hydrolysis fast enough to become a problem in practice. This means that it is feasible to thoroughly rinse whatever fluidic system is used to get rid of APS before sulfo-SMCC is introduced, to avoid that they mix in solution phase.

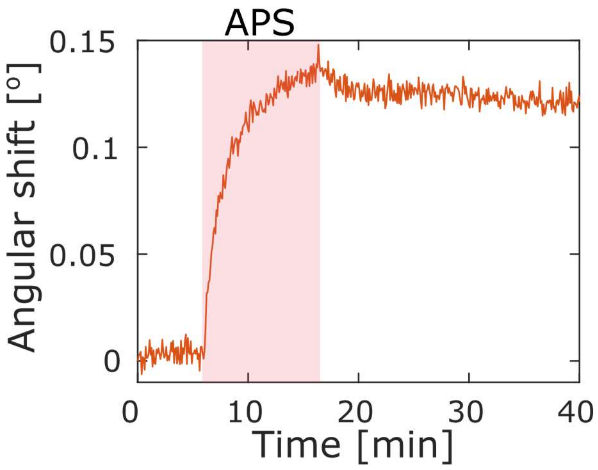

Figure S17 Binding of APS and rinsing in 95% ethanol monitored by SPR.

To confirm that the channels were not clogged, we also monitored the fluorescence intensity during the injections of avidin-FITC. Figure S18A shows that the protein is being transported by flow through both the microchannel and the nanochannels. The intensity is lower for the nanochannels partly due to their lower height and partly because they only occupy a fraction of the area. Still, the increase during the injection is clearly significant. As further confirmation, we could also observe a fluorescence increase in the outlet from the nanochannels during the injections. (See design in Figure S18B.)

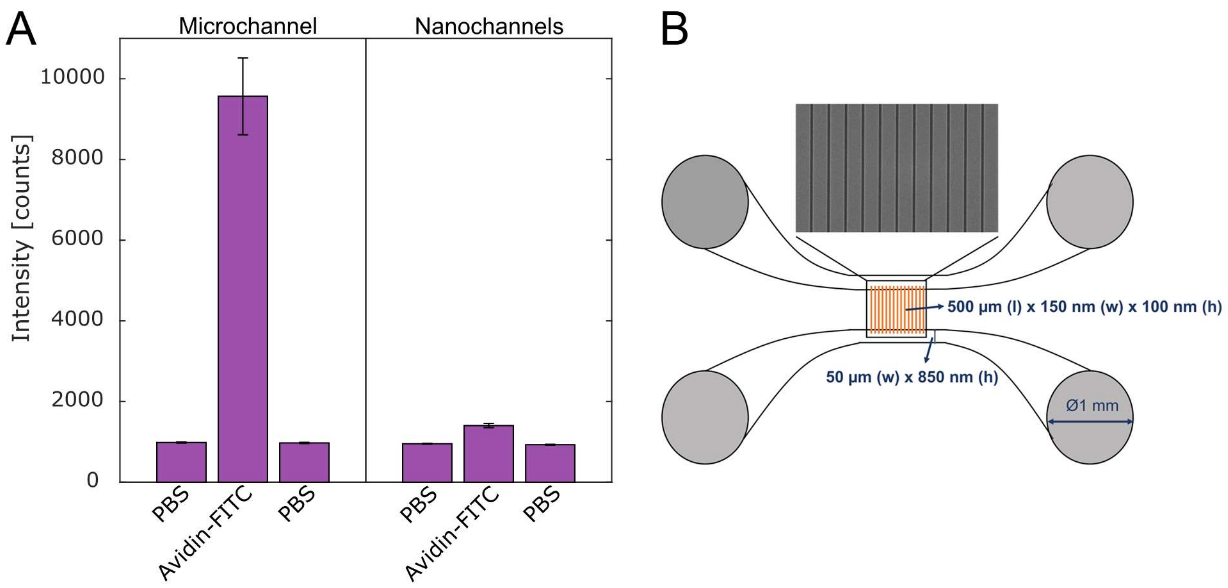

Figure S18 (A) Fluorescence intensity measured from the (passivated) channels before, during and after injection of the fluorescent protein Avidin-FITC. The error bars represent subsequent acquisitions. (B) Device design with two inlets and two outlets. The parallel nanochannels connect the two microfluidic channels.

Nanopore formation and conductance measurements

The nanopore diameter is normally estimated from the pore conductance G as:[S17]

$$d = \frac{G}{2\sigma} + \sqrt{\frac{G^2}{4\sigma^2} + \frac{4hG}{\pi\sigma}} \quad (S3)$$

Here h is the thickness of the membrane (20 nm from ellipsometry) and σ is the bulk conductivity of the electrolyte. We measured σ = 10.7 Sm^{-1} for the 1 M KCl solution using a CDM210 (MeterLab). Equation S3 gives a good measure of pore diameter under the assumption that that the shape is cylindrical. For the pore modified with PEG, the result should be interpreted as an effective diameter, representing a cylindrical opening containing only the electrolyte.

Fabrication by controlled dielectric breakdown is illustrated in Figure S19, where the sudden increase in current shows pore formation. To change the size of the pore, conditioning cycles were run using the protocol in the software and the change in diameter was monitored.

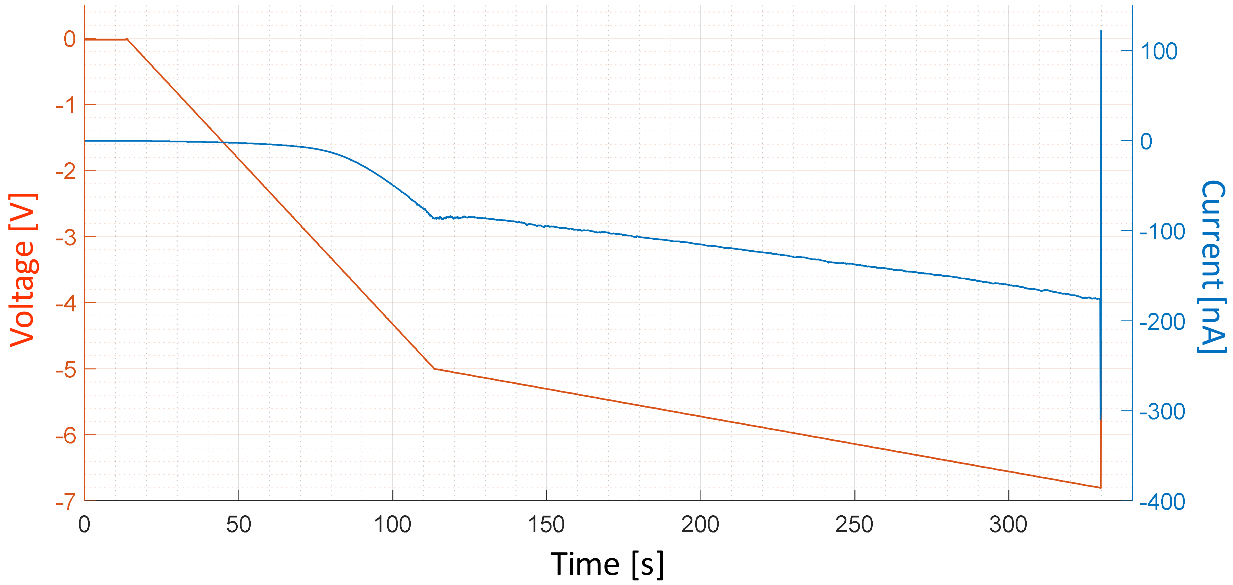

Figure S19 Example of controlled dielectric breakdown for nanopore formation. The pore is formed after ~330 s as evident by the sudden increase in current. The voltage is then switched off.

References

S1. Chen, S.-W.; Hong, T. T. A.; Chiang, C.-T.; Chau, L.-K.; Huang, C.-J., Versatile Thiol- and Amino-Functionalized Silatranes for in-situ Polymerization and Immobilization of Gold Nanoparticles. *Journal of the Taiwan Institute of Chemical Engineers* **2022**, *132*, 104129.
S2. Tabor, R. F.; Morfa, A. J.; Grieser, F.; Chan, D. Y. C.; Dagastine, R. R., Effect of Gold Oxide in Measurements of Colloidal Force. *Langmuir* **2011**, *27*, 6026-6030.
S3. Emilsson, G.; Schoch, R. L.; Feuz, L.; Hook, F.; Lim, R. Y. H.; Dahlin, A. B., Strongly Stretched Protein Resistant Poly(ethylene glycol) Brushes Prepared by Grafting-To. *ACS Applied Materials & Interfaces* **2015**, *7*, 7505-7515.
S4. Heinig, M. F.; Bastos da Silva Fanta, A.; Wagner, J. B.; Kadkhodazadeh, S., Aminopropylsilatrane Linkers for Easy and Fast Fabrication of High-Quality 10 nm Thick Gold Films on SiO2 Substrates. *ACS Applied Nano Materials* **2020**, *3*, 4418-4427.
S5. Miranda, A.; Martínez, L.; de Beule, P. A. A., Facile Synthesis of an Aminopropylsilane Layer on Si/SiO2 Substrates Using Ethanol as APTES Solvent. *MethodsX* **2020**, *7*, 100931.
S6. Okhrimenko, D. V.; Budi, A.; Ceccato, M.; Cardenas, M.; Johansson, D. B.; Lybye, D.; Bechgaard, K.; Andersson, M. P.; Stipp, S. L. S., Hydrolytic Stability of 3-Aminopropylsilane Coupling Agent on Silica and Silicate Surfaces at Elevated Temperatures. *ACS Applied Materials & Interfaces* **2017**, *9*, 8344-8353.
S7. Min, H.; Girard-Lauriault, P.-L.; Gross, T.; Lippitz, A.; Dietrich, P.; Unger, W. E. S., Ambient-Ageing Processes in Amine Self-Assembled Monolayers on Microarray Slides as Studied by ToF-SIMS with Principal Component Analysis, XPS, and NEXAFS Spectroscopy. *Anal. Bioanal. Chem.* **2012**, *403*, 613-623.
S8. Campbell, J. L.; Papp, T., Widths of the Atomic K–N7 Levels. *Atomic Data and Nuclear Data Tables* **2001**, *77*, 1-56.
S9. Andersson, J.; Ferrand-Drake del Castillo, G.; Bilotto, P.; Hook, F.; Valtiner, M.; Dahlin, A., Control of Polymer Brush Morphology, Rheology, and Protein Repulsion by Hydrogen Bond Complexation. *Langmuir* **2021**, *37*, 4943-4952.
S10. Ferrand-Drake del Castillo, G.; Emilsson, G.; Dahlin, A., Quantitative Analysis of Thickness and pH Actuation of Weak Polyelectrolyte Brushes. *The Journal of Physical Chemistry C* **2018**, *122*, 27516-27527.
S11. Emilsson, G.; Schoch, R. L.; Oertle, P.; Xiong, K.; Lim, R. Y. H.; Dahlin, A. B., Surface Plasmon Resonance Methodology for Monitoring Polymerization Kinetics and Morphology Changes of Brushes - Evaluated with Poly(N-isopropylacrylamide). *Applied Surface Science* **2017**, *396*, 384-392.
S12. Voinova, M. V.; Rodahl, M.; Jonson, M.; Kasemo, B., Viscoelastic Acoustic Response of Layered Polymer Films at Fluid-Solid Interfaces: Continuum Mechanics Approach. *Physica Scripta* **1999**, *59*, 391-396.
S13. Frykholm, K.; Muller, V.; Kk, S.; Dorfman, K. D.; Westerlund, F., DNA in Nanochannels – Theory and Applications. *Quarterly Reviews of Biophysics* **2022**, *55*, E12.
S14. Ying, Y.-L.; Hu, Z.-L.; Zhang, S.; Qing, Y.; Fragasso, A.; Maglia, G.; Meller, A.; Bayley, H.; Dekker, C.; Long, Y.-T., Nanopore-Based Technologies Beyond DNA Sequencing. *Nature Nanotechnology* **2022**, *17*, 1136-1146.
S15. Xue, L.; Yamazaki, H.; Ren, R.; Wanunu, M.; Ivanov, A. P.; Edel, J. B., Solid-State Nanopore Sensors. *Nature Reviews Materials* **2020**, *5*, 931-951.
S16. Eggenberger, O. M.; Ying, C.; Mayer, M., Surface Coatings for Solid-State Nanopores. *Nanoscale* **2019**, *11*, 19636-19657.

S17. Kwok, H.; Briggs, K.; Tabard-Cossa, V., Nanopore Fabrication by Controlled Dielectric Breakdown. *Plos One* **2014**, *9*, e92880.
S18. Vandenberg, E. T.; Bertilsson, L.; Liedberg, B.; Uvdal, K.; Erlandsson, R.; Elwing, H.; Lundstrom, I., Structure of 3-Aminopropyl Triethoxy Silane on Silicon Oxide. *Journal of Colloid and Interface Science* **1991**, *147*, 103-118.
S19. Lee, T.-J.; Chau, L.-K.; Huang, C.-J., Controlled Silanization: High Molecular Regularity of Functional Thiol Groups on Siloxane Coatings. *Langmuir* **2020**, *36*, 5935-5943.
S20. Huang, C. J.; Zheng, Y. Y., Controlled Silanization Using Functional Silatrane for Thin and Homogeneous Antifouling Coatings. *Langmuir* **2019**, *35*, 1662-1671.
S21. Zhu, M.; Lerum, M. Z.; Chen, W., How to Prepare Reproducible, Homogeneous, and Hydrolytically Stable Aminosilane-Derived Layers on Silica. *Langmuir* **2012**, *28*, 416-423.
S22. Lindberg, B. J.; Hamrin, K.; Johansson, G.; Gelius, U.; Fahlman, A.; Nordling, C.; Siegbahn, K., Molecular Spectroscopy by Means of ESCA II. Sulfur Compounds. Correlation of Electron Binding Energy with Structure. *Physica Scripta* **1970**, *1*, 286.
S23. Svirelis, J.; Andersson, J.; Stradner, A.; Dahlin, A., Accurate Correction of the “Bulk Response” in Surface Plasmon Resonance Sensing Provides New Insights on Interactions Involving Lysozyme and Poly(ethylene glycol). *ACS Sensors* **2022**, *7*, 1175-1182.
S24. Shafer, D. E.; Inman, J. K.; Lees, A., Reaction of Tris(2-carboxyethyl)phosphine (TCEP) with Maleimide and α-haloacyl Groups: Anomalous Elution of TCEP by Gel Filtration. *Analytical Biochemistry* **2000**, *282*, 161-164.
S25. Burns, J. A.; Butler, J. C.; Moran, J.; Whitesides, G. M., Selective Reduction of Disulfides by Tris(2-carboxyethyl)phosphine. *The Journal of Organic Chemistry* **1991**, *56*, 2648-2650.
